# Nano‐in‐Microparticles for Aerosol Delivery of Antibiotic‐Loaded, Fucose‐Derivatized, and Macrophage‐Targeted Liposomes to Combat Mycobacterial Infections: In Vitro Deposition, Pulmonary Barrier Interactions, and Targeted Delivery

**DOI:** 10.1002/adhm.202102117

**Published:** 2022-02-18

**Authors:** Benedikt C. Huck, Durairaj Thiyagarajan, Aghiad Bali, Annette Boese, Karen F. W. Besecke, Constantin Hozsa, Robert K. Gieseler, Marcus Furch, Cristiane Carvalho‐Wodarz, Franziska Waldow, Dominik Schwudke, Olga Metelkina, Alexander Titz, Hanno Huwer, Konrad Schwarzkopf, Jessica Hoppstädter, Alexandra K. Kiemer, Marcus Koch, Brigitta Loretz, Claus‐Michael Lehr

**Affiliations:** ^1^ Department of Drug Delivery Helmholtz Institute for Pharmaceutical Research Saarland Campus E8.1 Saarbrücken 66123 Germany; ^2^ Department of Pharmacy Helmholtz Institute for Pharmaceutical Research Saarland Saarland University Campus E8 1 Saarbrücken 66123 Germany; ^3^ Department of Anti‐infective Drug Discovery Helmholtz Institute for Pharmaceutical Research Saarland Campus E8 1 Saarbrücken 66123 Germany; ^4^ Rodos Biotarget GmbH Hannover 30625 Germany; ^5^ Laboratory of Immunology and Molecular Biology and Department of Internal Medicine University Hospital Knappschaftskrankenhaus Bochum Ruhr University Bochum Bochum 44892 Germany; ^6^ Research Center Borstel Leibniz Lung Center Borstel 23845 Germany; ^7^ German Center for Infection Research Thematic Translational Unit Tuberculosis Partner Site Hamburg‐Lübeck‐Borstel‐Riems Braunschweig 38124 Germany; ^8^ German Center for Lung Research (DZL) Airway Research Center North (ARCN) Kiel Nano Surface and Interface Science KiNSIS Kiel University Kiel 24118 Germany; ^9^ Chemical Biology of Carbohydrates (CBCH) Helmholtz‐Institute for Pharmaceutical Research Saarland (HIPS) Helmholtz Center for Infection Research Saarbrücken 66123 Germany; ^10^ Department of Chemistry Saarland University Saarbrücken 66123 Germany; ^11^ Deutsches Zentrum für Infektionsforschung (DZIF) Hannover‐Braunschweig site Braunschweig 38124 Germany; ^12^ Cardiothoracic Surgery Heart Center Voelklingen Völklingen 66333 Germany; ^13^ Department of Anaesthesia and Intensive Care Klinikum Saarbrücken gGmbH Saarbrücken 66119 Germany; ^14^ Pharmaceutical Biology Saarland University Campus C2 3 Saarbrücken 66123 Germany; ^15^ INM – Leibniz Institute for New Materials Campus D2 2 Saarbrücken 66123 Germany; ^16^ Present address: Solmic BioTech GmbH Düsseldorf 40225 Germany; ^17^ Present address: Siegfried AG Hameln Hameln 31789 Germany; ^18^ Present address: Biolife Holding AG Heidelberg 69126 Germany

**Keywords:** air–liquid interfaces, bedaquiline, liposomal dry powders, particle tracking, pulmonary surfactants

## Abstract

Nontuberculous mycobacterial infections rapidly emerge and demand potent medications to cope with resistance. In this context, targeted loco‐regional delivery of aerosol medicines to the lungs is an advantage. However, sufficient antibiotic delivery requires engineered aerosols for optimized deposition. Here, the effect of bedaquiline‐encapsulating fucosylated versus nonfucosylated liposomes on cellular uptake and delivery is investigated. Notably, this comparison includes critical parameters for pulmonary delivery, i.e., aerosol deposition and the noncellular barriers of pulmonary surfactant (PS) and mucus. Targeting increases liposomal uptake into THP‐1 cells as well as peripheral blood monocyte‐ and lung‐tissue derived macrophages. Aerosol deposition in the presence of PS, however, masks the effect of active targeting. PS alters antibiotic release that depends on the drug's hydrophobicity, while mucus reduces the mobility of nontargeted more than fucosylated liposomes. Dry‐powder microparticles of spray‐dried bedaquiline‐loaded liposomes display a high fine particle fraction of >70%, as well as preserved liposomal integrity and targeting function. The antibiotic effect is maintained when deposited as powder aerosol on cultured *Mycobacterium abscessus*. When treating *M. abscessus* infected THP‐1 cells, the fucosylated variant enabled enhanced bacterial killing, thus opening up a clear perspective for the improved treatment of nontuberculous mycobacterial infections.

## Graphical abstract

Nano‐in‐microparticles for aerosol delivery of antibiotic‐loaded and fucose‐derivatized liposomes are developed to combat mycobacterial infections. In vitro studies with various phagocytic cell types reveal the benefits of macrophage‐targeted pulmonary delivery, which oftentimes is complicated by the interaction of antibiotics with pulmonary barriers, if delivered in nonencapsulated form. The inclusion of pulmonary mucus and surfactant into such models helps to estimate key factors that may influence therapeutic outcomes.

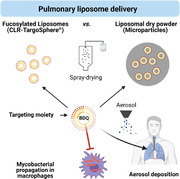

## 1 Introduction

According to the WHO, mycobacterial infections are among the top ten causes of mortality worldwide with 7.1 million new infections and 1.4 million deaths in 2020.^[^ ^1^ ^]^ Given the increase of antibiotic resistance, the treatment of such intracellular infections calls for improved medications and delivery approaches.^[^ ^2^, ^3^ ^]^ Poor drug permeability and systemic availability of orally administered antimycobacterial compounds including bedaquiline (BDQ) and levofloxacin (LVX) complicate the situation.^[^ ^4^ ^]^ Therefore, therapeutic approaches that achieve high antibiotic concentrations at the target site are required. Targeted, local pulmonary delivery may complement conventional oral therapy, that is associated with high doses, long treatment periods, and severe side effects.^[^ ^2^, ^5^ ^]^ Here, the nature of the drug delivery system is essential as it must safely and efficiently encapsulate the antibiotic cargo, enable to reach the deeper lung, enter the target cells, and release the antibiotic into the appropriate intracellular compartments.^[^ ^6^ ^]^ Further challenges result from interactions with the sophisticated pulmonary defense mechanisms, including renewable cellular (epithelial cells) and noncellular (pulmonary mucus and surfactant) barriers.^[^ ^7^ ^]^ Liposomal drug carriers have evolved as potent delivery system by increasing both solubility and permeability of the active compound, particularly for intracellular infections.^[^ ^8^ ^]^ The high cellular uptake rate of liposomes further benefits from controlled, targeted delivery to macrophages via receptor‐mediated endocytosis in vitro and in vivo.^[^ ^9^ ^]^ By specifically addressing the macrophage mannose receptor (CD206) using fucosylated TargoSphere liposomes, Duran et al. already demonstrated an increased cellular uptake and efficacy of such LVX‐loaded nanocarriers. Receptor‐mediated endocytosis and intracellular routing thus direct the encapsulated antibiotics to acidic compartments where they can efficiently kill bacteria residing therein.^[^ ^10^ ^]^

There are, however, several obstacles when delivering liposomes to the deep lungs, i.e., first, the branching lung architecture and, second, the lungs’ protective barrier mechanisms that challenge deep lung deposition of nanosized drug carriers.^[^ ^11^ ^]^ The specific infection pathway of *Mycobacterium abscessus* is not fully understood but may include colonization of the respiratory mucosa and the resistance against intracellular killing within macrophages, in particular for patients with chronic lung diseases (COPD, bronchiectasis, cystic fibrosis) or in immunocompromised patients. The clinical manifestations of *M. avium* and *M. tuberculosis* infections show similarities and can lead to granuloma formation and/or colonization of the lung airways as biofilms as enduring, difficult‐to‐eradicate infections.^[^ ^12^ ^]^ The main targets likely are the granulomas and/or mucus microaggregate biofilms located in the deep lung region and the respiratory bronchioles. Thus, characterizing the interaction of aerosolized liposomal dry powders with pulmonary mucus will help to predict their potential for lung applications.^[^ ^13^ ^]^ In the distal airways at the alveolar air–blood epithelium, pulmonary surfactant (PS) lines the interface. This hydrophobic, lipid‐rich material consists of four major surfactant proteins (SP), i.e., hydrophilic SP‐A and SP‐D and hydrophobic SP‐B and SP‐C.^[^ ^14^, ^15^ ^]^ Pulmonary absorption, clearance, and drug release are influenced by the bio–nano interactions with the PS that determine the fate of inhaled nanomaterials in the lungs. Depending on the type and surface properties of nanomaterials, contradicting observations range from increased uptake by opsonization through surfactant proteins to decreased uptake in case of silica nanoparticles.^[^ ^16^ ^]^ Therefore, to evaluate possible effects of PS on the liposomes’ integrity and uptake, a case‐by‐case study for newly developed liposomes or other nanomaterials is crucial. Broichsitter et al. demonstrated that the release of 5‐carboxyfluorescein from liposomes upon contact with pulmonary surfactant (Alveofact) is altered both in vitro and in vivo.^[^ ^17^ ^]^ Another aspect to be considered is the ability of fluoroquinolones including LVX to rapidly permeate the alveolar epithelium requiring formulation approaches to enhance lung residence time.^[^ ^18^ ^]^

A process often heeded only late in the development process is the demand for an inhalable dosage form with optimized aerodynamic properties and sufficient storage stability. The latter is particularly relevant for mycobacterial infections that mainly occur in low‐income countries with inappropriate storage conditions. The intricacy of developing dry powders is to maintain the integrity of both the liposome and the cargo during production and storage.^[^ ^19^ ^]^ Although liposomes are promising delivery systems, the removal of bulk water during a drying procedure may lead to the formation of their thermodynamically unstable multilamellar states when compared to solid or polymeric nanoparticles.^[^ ^20^ ^]^ The removal of water, however, is crucial to maintain long‐term colloidal and microbiological stability. Therefore, the optimization of methodical parameters is crucial to obtain dry powders with a high fine particle fraction, good flowability, and sufficient drug load while maintaining liposomal integrity.^[^ ^21^ ^]^ Recent publications using lactose and leucine as excipients have described the successful generation of liposomal dry powders loaded with dapsone for inhalation against pulmonary infections.^[^ ^22^ ^]^ However, these nanocarriers did not contain components for active targeting. The stability and interaction potential of a nanocarrier depends on its surface properties, which might be altered by the nature and amount of targeting ligand. Consequently, testing the targeted dry‐powder formulation under relevant deposition conditions is required to evaluate the efficacy of the final product.

**Scheme 1:**
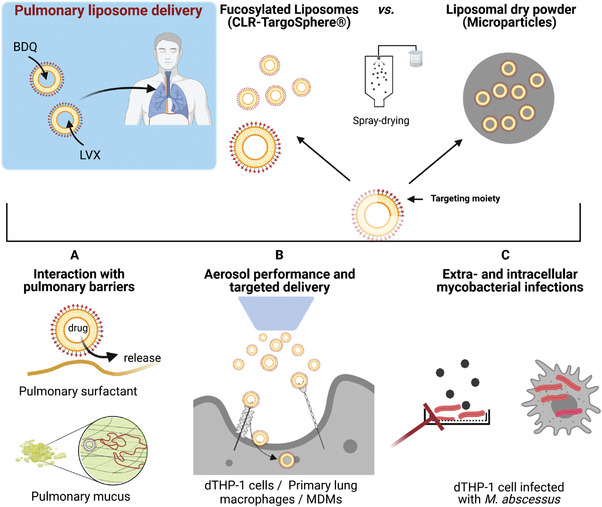
Biological barriers in pulmonary drug delivery. Targeted liposomes and liposomal dry‐powder formulations loaded with bedaquiline (BDQ) or levofloxacin (LVX), respectively, were developed to overcome such barriers. A) Interaction of liposomes with epithelial cells and pulmonary macrophages in the presence of mucus and surfactant. B) Receptor‐mediated internalization of targeted liposomes into myeloid cells under submerged and air–liquid interface conditions and in the presence of pulmonary surfactant. C) Assessing antimycobacterial activity against extracellular and intracellular *Mycobacterium abscessus*. Abbreviations: MDMs: Peripheral blood monocyte‐derived macrophages, dTHP‐1: differentiated THP‐1 cells.

Here, we investigated whether the advantage of C‐type lectin receptor (CLR‐)TargoSphere liposomes for targeted delivery of BDQ to monocytes—the mycobacterial habitat—is maintained after their conversion into a respirable dry‐powder aerosol formulation. The latter was obtained by spray‐drying of liposomes, together with lactose and leucine, followed by a thorough characterization of the resulting microspheres as to their aerodynamic properties, liposomal integrity, drug load, and efficacy against *M. abscessus*. We further considered it as important to study the multiple interactions of such complex carrier systems with the lungs’ biological barriers at different levels under controlled conditions in vitro. To investigate the interaction with macrophages in the presence of a lung lining fluid, we used different human cells (the differentiated THP‐1 cell line, referred to as dTHP‐1, as well as primary human peripheral blood‐derived and pulmonary macrophages) and the commercially available phospholipid preparation, Alveofact, as suitable lung surfactant substitute.^[^ ^23^ ^]^ We further investigated the interaction of deposited aerosol particles with human tracheobronchial mucus. For release and permeability studies, LVX was included as a control, and to allow for some conclusions on the importance of drug properties in such interactions always by systematically comparing plain versus targeted liposomes before and after spray‐drying. Finally, successful drug delivery and efficacy was assessed by the killing efficacy of *M. abscessus* in culture by powder aerosol deposition as well as by the killing of intracellular bacteria after infection of dTHP‐1 cells. The overall concept of the study is summarized in **Scheme** **1**.

## 2 Results

### 2.1 Characterization and Physicochemical Properties of Liposome Variants

Both LVX‐ and BDQ‐loaded liposomes displayed homogenous size distributions with polydispersity index (PDI) values of <0.1 and a size range of 90–110 nm. Liposomes had a negative *ζ*‐potential with higher values for LVX‐ than for BDQ‐loaded liposomes (−40 mV vs −14 mV). The aforementioned parameters remained unchanged regardless of the presence or absence of the fucose ligand for targeting of relevant macrophage CLRs such as CD206 and CD209. **Table** **1** summarizes the physicochemical properties including the encapsulation efficiency and drug load of the respective formulations.

**Table 1:** Size, PDI, *ζ*‐potential, drug load and encapsulation efficacy of TargoSphere liposomes (*n* = 3–5 batches)

Sample	Targeting	Size [nm]	PDI	*ζ*‐potential [mV]	Encapsulation efficacy [%]	Loading capacity [%]
TS_Levo	No	98.7 ± 3.0	0.1 ± 0.03	−39.6 ± 2.6	80.2 ± 6.9	17.2 ± 4.4
TS_Levo	Yes	110.2 ± 1.4	0.04 ± 0.01	−39.4 ± 3.5	66.0 ± 21.4	14.5 ± 5.1
TS_BDQ	No	97.6 ± 0.2	0.07 ± 0.01	−15.8 ± 1.3	98.2 ± 3.4	6.4 ± 1.3
TS_BDQ	Yes	89.2 ± 0.4	0.08 ± 0.01	−13.1 ± 1.2	98.0 ± 1.7	7.6 ± 2.5

**Figure 1:**
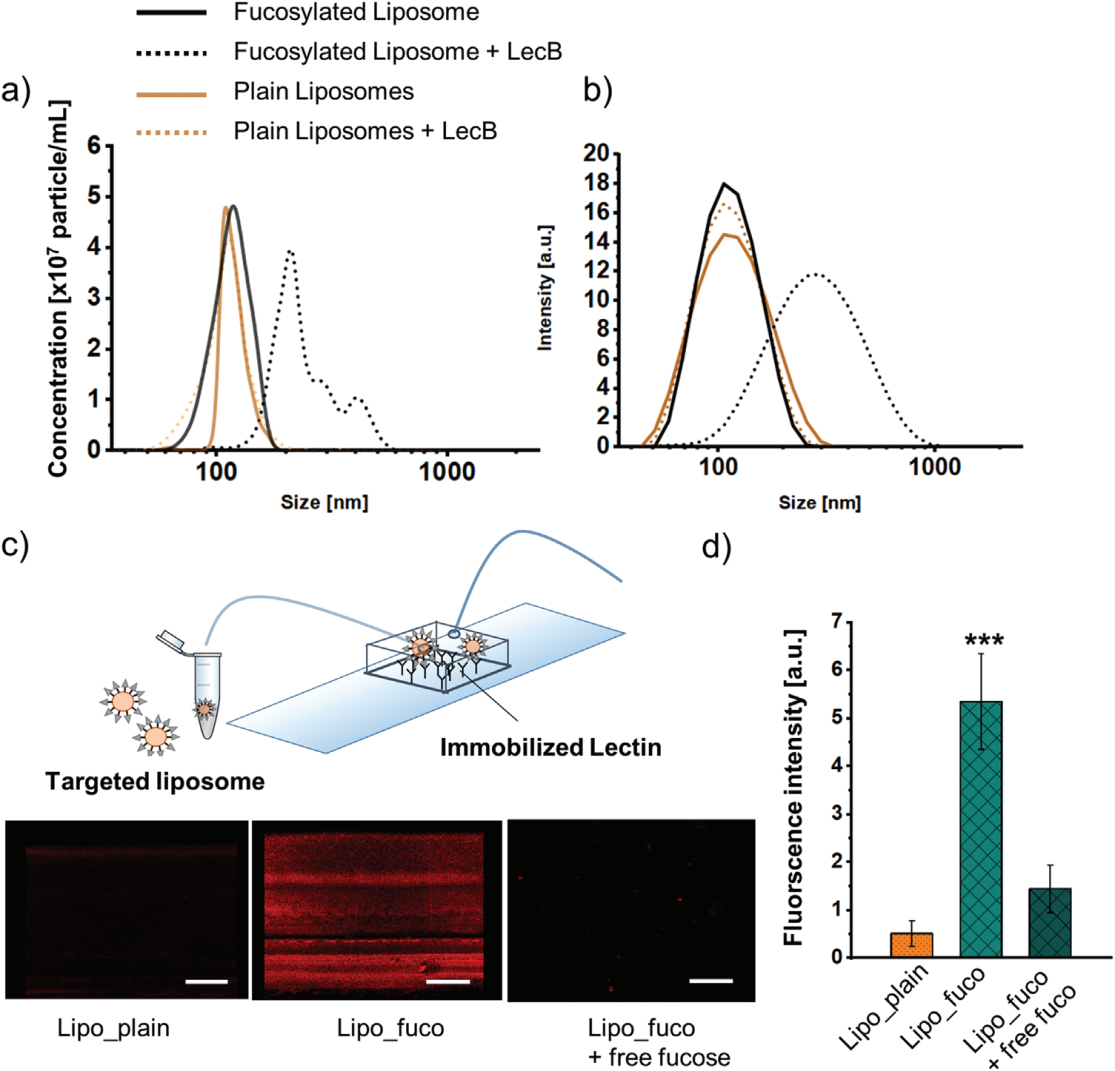
Size distribution of fucosylated liposomes in the presence of LecB after overnight incubation at a 2:1 molar ratio measured by a) particle tracking analysis and b) dynamic light scattering. c) Microfluidic assay with LecB covalently attached to the bottom of the flow chamber. Following a 10‐min flush with TargoSphere liposomes and a 30‐min rest, excess CLR‐TargoSpheres were removed by flushing the chamber for 15 min with buffer. Images are representative for nontargeted liposomes (left), targeted liposomes (center), and targeted liposomes after an additional flushing step with competitive inhibitor (right). d) Quantification of fluorescence intensity. Error bars indicate means ± SD (*n* = 12–15, *N* = 3). Significance was defined as *** *(p* < 0.001).

To verify the presence of the targeting ligands on the surface of liposomes, lectin binding assays were performed for BDQ‐loaded liposomes. First, overnight incubation of liposomes with the fucose‐specific *Pseudomonas aeruginosa* lectin, LecB, led to a significant shift of the size distribution to higher values as observed by nanoparticle tracking (**Figure** **1**). This was only the case for fucosylated, but not plain liposomes, thus indicating specific lectin‐mediated liposome clustering. Next, the binding of perfused liposomes to LecB immobilized on the bottom of a microfluidic chamber was assessed.^[^ ^24^ ^]^ Upon adding targeted liposomes for 30 min, liposomes tightly bound to the bottom of the glass slides were visualized by confocal microscopy. The resulting ten‐fold difference in fluorescence intensity revealed the specific attachment of targeted liposomes to the immobilized lectin. Subsequent equilibration with buffer did not detach the TargoSphere liposomes, whereas the addition of a competitive inhibitor (i.e., 250 × 10^−3^ m l‐fucose solution) removed most of them.

### 2.2 Drug Release and Permeability in Lung‐Relevant Media and Cells

**Figure 2:**
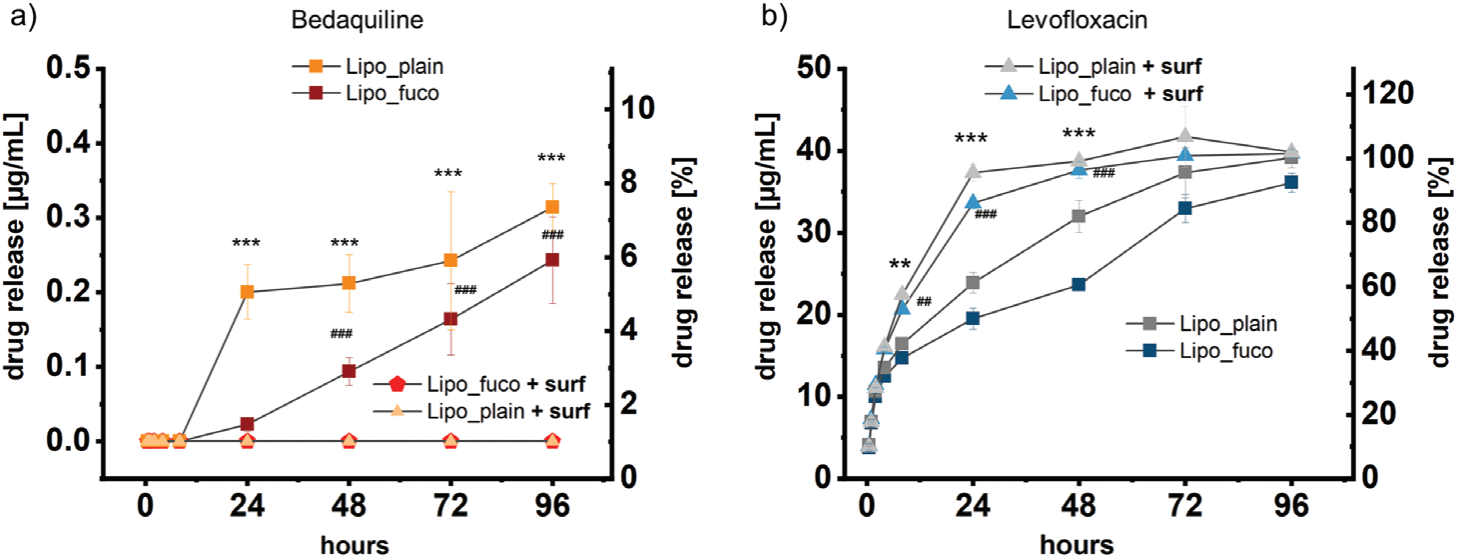
Release profiles of a) bedaquiline (BDQ) and b) levofloxacin (LVX) from fucosylated (Lipo_fuco) and plain liposomes (Lipo_plain) in the presence and absence of pulmonary surfactant Alveofact, respectively. LVX was released more rapidly in the presence of pulmonary surfactant (PS) and compared to BDQ. The release of BDQ did not exceed 10% after 96 h and was not detectable when PS was present in the donor medium. Error bars represent means ± SD (*n* = 9, *N* = 3). Significance was defined as ***/^###^ (*p* < 0.001) and **/^##^ (*p* < 0.005).

To determine the drug release from liposomes under lung‐relevant conditions, the clinically used PS Alveofact was added to the respective release medium. **Figure** **2**a shows that >80% of the more hydrophilic antibiotic, LVX, is released in the presence of PS already after 24 h, whereas in the absence of PS a similar amount was released after 72 h. In contrast, the concentration of the strongly hydrophobic and practically water‐insoluble BDQ even after 96 h never exceeded 10% of the total drug load. (Figure 2b). After the same time, the BDQ amount in the acceptor compartment was strikingly below the detection limit when in the presence of PS. In spite of a slightly different lipid composition of the two formulations, the association of the drug within the hydrophilic core (for LVX) or the hydrophobic bilayered membrane (for BDQ) was comparable. Thus, release studies in lung‐mimicking conditions revealed prominent differences depending on the nanomedical formulation (here: the liposomal composition and the biochemical nature of the encapsulated drug). Interestingly, liposomal encapsulation of LVX also reduced the membrane permeability when compared to the free drug, thereby potentially increasing the pulmonary residence time (Figure S1a,b, Supporting Information).

### 2.3 Cellular Liposome Uptake by Macrophages under Submerged and Air–Liquid Interface Conditions

**Figure 3:**
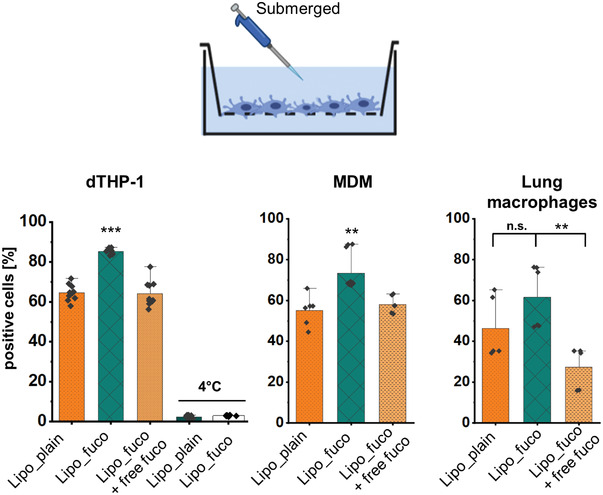
Quantification of liposome uptake by flow cytometry under submerged conditions at a concentration of 50 µg mL^−1^ after 2 h incubation at 37 °C in volumes of 300 µL per condition. Fucosylated liposomes showed a higher uptake than plain liposomes. Increased uptake into human dTHP‐1 cells, peripheral blood monocyte‐derived macrophages (MDM) and lung tissue macrophages was reduced in the presence of free l‐fucose (10 × 10^−3^ m) as a competitive inhibitor. At 4 °C, the internalization was drastically reduced due to the inhibition of active uptake, thus indicating the proportion of cell‐associated liposomes in this specific case. Data represent means ± SD (*n* = 6–9, *N* = 3 for dTHP‐1 and MDM, and *n* = 3–5 with *N* = 2 for lung macrophages). Significance was defined as *** (*p* < 0.001), ** (*p* < 0.005), and * (*p* < 0.05).

The effect of fucose‐functionalization on the cellular internalization was investigated on three different cell types, i.e., the differentiated human acute monocytic leukemia‐derived cell line, dTHP‐1, as well as human primary tissue‐derived alveolar‐ and peripheral blood monocyte‐derived macrophages (MDM). The selected cells, in particular the primary ones, express a high level of C‐type lectin receptors, including the CD206 receptor, which facilitate mycobacterial internalization by myeloid lineage‐derived cells.^[^ ^25^ ^]^ Similarly, the internalization of targeted liposomes by such cells is intended to shuttle the antibiotic cargo into their endosomal compartments. All types of macrophages showed an increase in uptake of PE‐Texas red‐labeled fucosylated liposomes after 2 h incubation at 37 °C under submerged conditions. Plain liposomes and cells pre‐incubated with 10 × 10^−3^ m l‐fucose solution displayed 20% lower uptake than fucosylated TargoSphere liposomes (**Figure** **3** and Figure S2, Supporting Information). The same tendency was observed for both dTHP‐1 and primary cells, although the latter showed a higher CD206 expression (Figure S3, Supporting Information).

To demonstrate active phagocytosis, dTHP‐1 cells were additionally incubated at 4 °C, whereupon <5% of the cells showed uptake/association. This proportion of cells might indicate the extent of liposomal binding to the cell surface.

**Figure 4:**
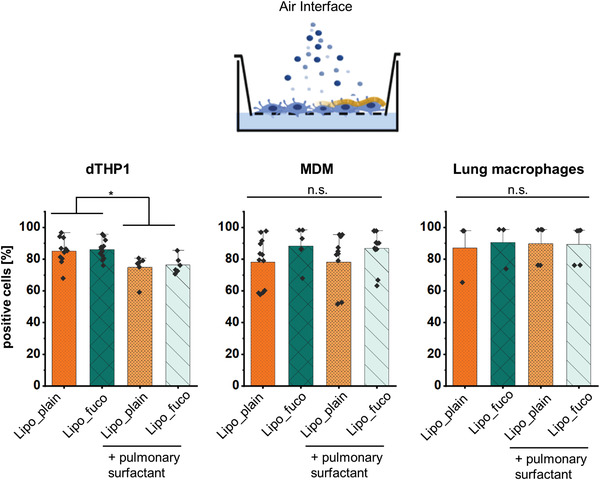
Quantification of liposome uptake by flow cytometry after aerosol deposition at a dose of 15 µg/well after 2‐h incubation at 37 °C in the presence or absence of pulmonary surfactant, Alveofact, respectively. When pulmonary surfactant was present in the interface, the uptake into dTHP‐1 cells was reduced, which was not observed for blood‐monocyte derived macrophages (MDM) and macrophages from human lung tissue. Data represent mean ± SD (*n* = 6–9, *N* = 3 for dTHP‐1 and MDM and *n* = 3–5 with *N* = 2 for lung macrophages). Significance was defined as * (*p* < 0.05).

Aerosolization of liposomes on cells that were transferred to air–liquid interface allows for a simulation of pulmonary deposition in the presence or absence of pulmonary surfactant (**Figure** **4**). When depositing the same dose using a vibrating mesh nebulizer, the number of positive cells generally increased when compared to submerged conditions. As the overall liposome uptake under air‐liquid interface conditions was high, no surplus uptake of fucose‐targeted liposomes was observed. The addition of PS to the interface did alter the uptake behavior in the case of dTHP‐1 cells, attributable to a lower fluctuation in uptake compared to primary cells, preventing to assess such deviations in the latter.

### 2.4 Development of BDQ‐Loaded Microparticles by Spray‐Drying

**Figure 5:**
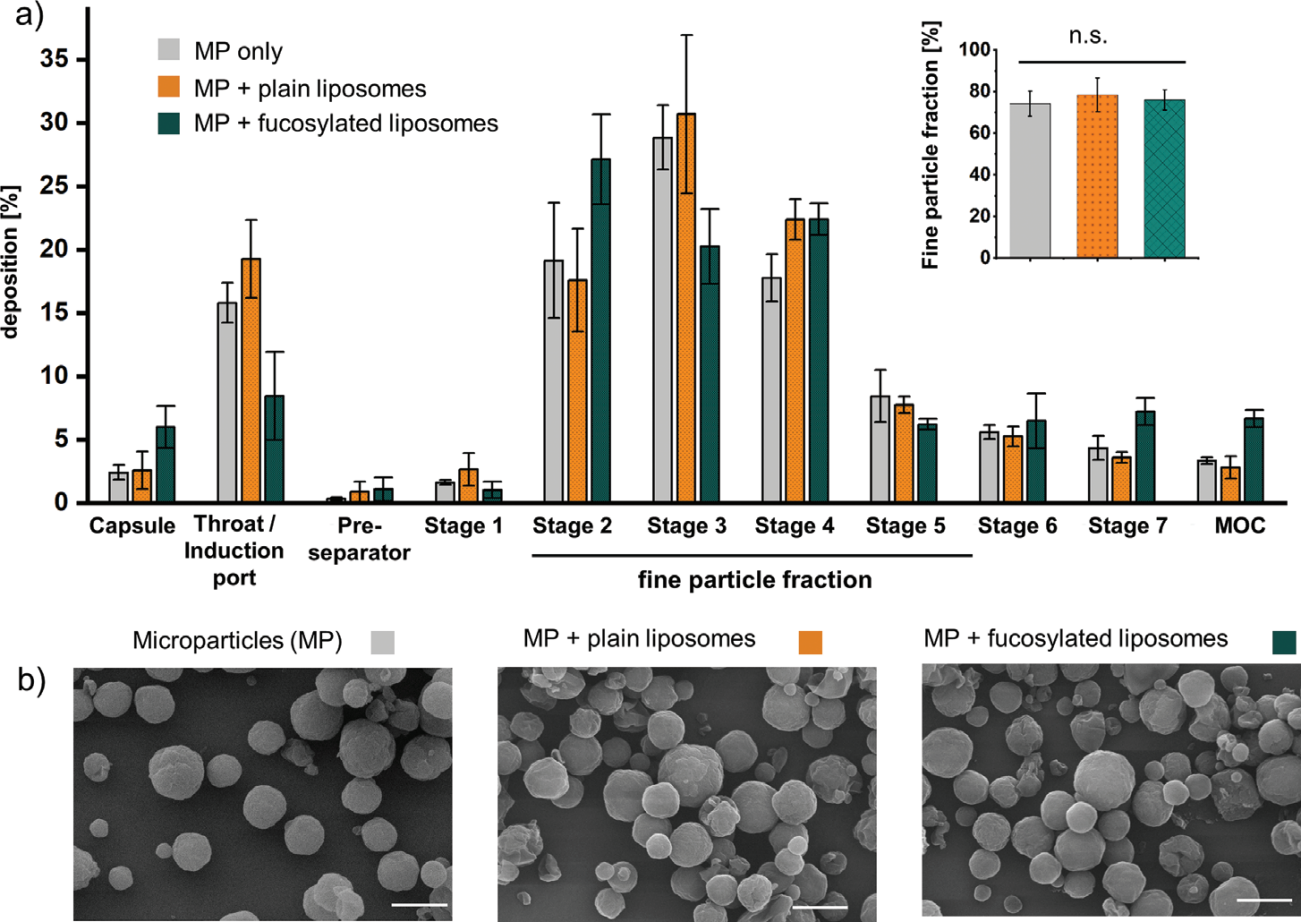
a) Aerodynamic properties of microparticles and their deposition in different stages of the next‐generation impactor and b) scanning electron microscopy images of empty lactose‐leucine microparticles (MP) and of MP containing fucosylated or plain liposomes, respectively. No differences in the fine‐particle fraction were found between the different MP variants. Scale bars: 2 µm. Data represent means ± SD from three independent replicates (*n* = 9, *N* = 3). Cut‐off sizes for the Next Generation Impactor at a flow rate of 60 L min^−1^ are 8.06, 4.46, 2.82, 1.66, 0.94, 0.55, and 0.34 µm for stages 1–7, and MOC for micro‐orifice collector, respectively.^[^ ^27^ ^]^

To produce a nano‐in‐micro system for aerosol delivery to the lung, lactose‐leucine‐based microparticles containing plain or fucosylated liposomes were produced by spray‐drying. Lactose and leucine were selected as well‐established excipients for dry‐powder formulations.^[^ ^26^ ^]^ When the concentrations of lactose and leucine were optimized to 2.5% or 1% w/v, respectively, stable microparticles with a high fine‐particle fraction were achieved. To determine its aerodynamic properties after adding BDQ‐loaded liposomes, the microparticles were investigated using a next‐generation impactor. At a flow rate of 60 L min^−1^, all investigated microparticles preferably deposited in stages 2–5 of the inertial impactor, which is equivalent to the fraction with a mass median aerodynamic diameter (MMAD) suitable to reach the deeper lungs. The amount of powder left in the capsule was <10%. However, there was a portion of 15%–20% deposited in the L‐shaped induction port that reflects the amount deposited in the throat (**Figure** **5**a). Yet, the fine‐particle fraction (equivalent to stages 2–5) was >70%, and it was preserved when adding either fucosylated or plain liposomes to the microparticles. Their volume mean diameter determined by static light scattering was determined as 2.6–3.0 µm, and all microparticles exhibited a spherical appearance and smooth surface morphology (Figure 5b and Figure S5, Supporting Information).

**Figure 6:**
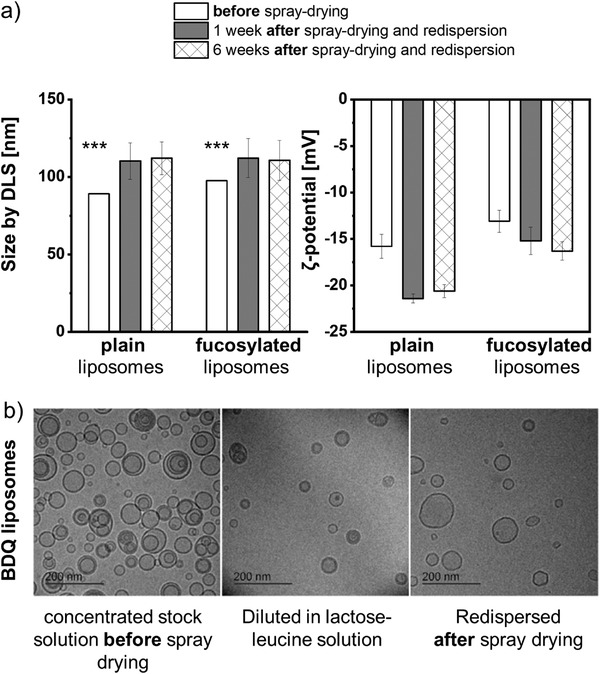
a) The initial size of the liposomes increases during spray‐drying and remains constant upon six weeks of storage. b) BDQ‐loaded liposomes before and after spray‐drying (and redispersion) have similar sizes with intact lipid bilayers as observed by cryo‐transmission electron microscopy. Data represent means ± SD (*n* = 6–8, *N* = 3) with significance defined as *** (*p* < 0.001).

Maintaining the integrity of the spray‐dried liposomes was essential to exert a proper functionality. This includes the liposomes’ size, shape, drug load, and active targeting. Immediately after spray‐drying, a 20‐nm size increase was observed for both plain and fucosylated liposomes; this size did not change over a test period of six weeks (**Figure** **6**a). The negative *ζ*‐potential of −14 mV only slightly increased after spray‐drying, which may contribute to stabilizing the liposomes at higher concentrations. The amount of BDQ retained in the microparticles determined by LC‐MS/MS was 1.03 ± 0.16 µg mg^−1^ of microparticles (dry powder) for plain and 1.00 ± 0.19 µg mg^−1^ microparticles for fucose‐targeted liposomes.

For more close inspection, cryogenic transmission microscopy images of TargoSphere liposomes from the initial batch (not spray‐dried), in lactose‐leucine solution (not spray‐dried) and after spray‐drying and redispersion were obtained (Figure 6b). Before and after spray‐drying, spherical liposomes with visibly intact membranes in a size range of <200 nm were observed. Liposome numbers were highest for those made from the initial batch with no additional dilution. The distribution of PE‐Texas red‐labeled liposomes within microparticles was further evaluated by confocal microcopy (Figure S6, Supporting Information).

### 2.5 Assessment of Targeting Function and Mucus Interaction after Spray‐Drying

**Figure 7:**
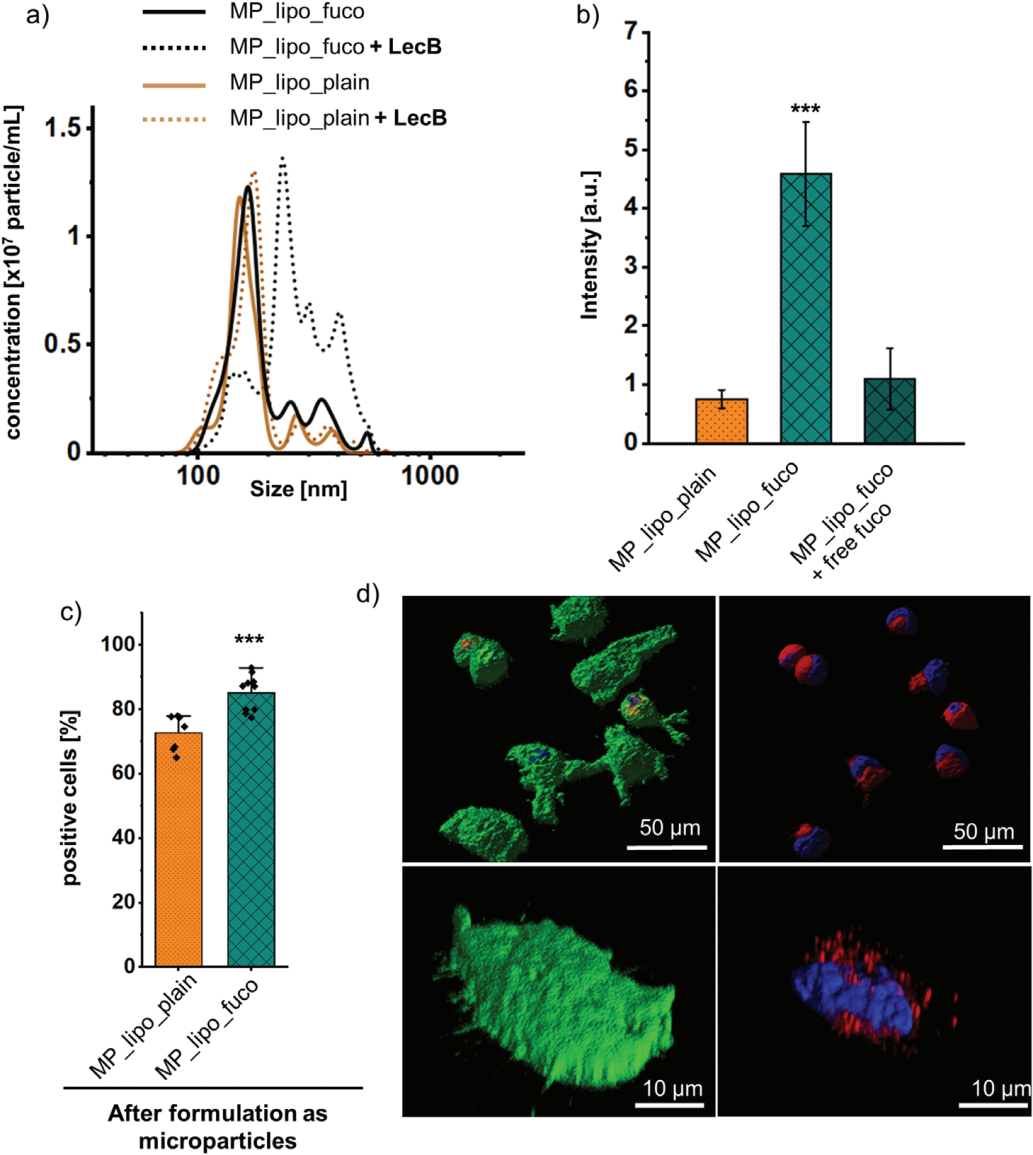
Targeting function of liposomes is preserved after spray‐drying as demonstrated by the a) LecB binding assay and b) in microfluidic chambers. c) Uptake of plain (MP_lipo_plain) and fucosylated (MP_lipo_fuco) BDQ‐loaded nano‐in‐microparticles following redispersion. Uptake after 2‐h incubation at 37 °C by dTHP‐1 cells evidenced by flow cytometry confirmed that active targeting was preserved. d) Representative confocal images analyzed for dissolved dry powders containing fucosylated liposomes show the intracellular localization of liposomes. Nuclei were stained with DAPI (blue), actin with phalloidin (green), and liposomes with PE‐Texas red. Data represent means ± SD (*n* = 9, *N* = 3) with significance defined as *** (*p* < 0.001).

After successful spray‐drying of BDQ‐loaded liposomes, the functionality of fucose‐targeting was explored. By applying the LecB binding assays already described in context with nonspray‐dried liposomes, a LecB‐mediated size increase and specific binding of fucosylated liposomes to immobilized LecB was observed (**Figure** **7**a,b). In addition, the percentage of PE‐Texas red positive dTHP‐1 cells treated with microparticles consisting of fucosylated liposomes was higher compared to their plain counterparts as assessed by flow cytometry (Figure 7c), thus indicating that the targeting function is preserved after spray‐drying. Next, confocal microscopy was applied to validate the efficiency of liposome internalization (Figure 7d). The images obtained for microparticles of fucosylated liposomes clearly depict their presence within the cells that are framed by the phalloidin‐stained actin‐rich filaments of the cell membrane (green, Figure 7d).

**Figure 8:**
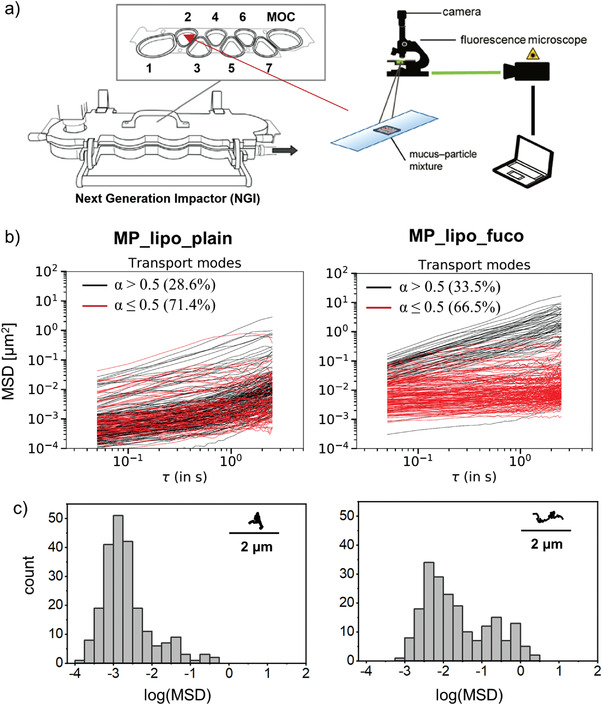
a) A slide with deposited tracheobronchial mucus was placed into stage 2 of the next generation impactor and analyzed by video microscopy after deposition of the dry powder to study the mobility of plain (MP_lipo_plain) and fucosylated (MP_lipo_fuco) liposomes released from microparticles. b) The slope of individual mean squared displacement (MSD) curves discriminates between mobile (black line, *α* > 0.5) and immobile (red line, *α* ≤ 0.5) particles. Percentages indicate the mobile/immobile fraction of all tracked particles. c) The distribution of log (MSD) values at a timescale of *t* = 0.5 s indicates a higher mobility of fucosylated liposomes, as also signified in the representative trajectory. Three independent experiments were performed with at least >150 particles/frame.

As considerable amounts of dry powder are lost in the small airways due to its interaction with pulmonary mucus, multiple particle tracking of liposomal dry powders was applied to assess the extent of liposomes that bind to the mucus layer. Therefore, microparticles were deposited on a glass slide that was covered with a small volume of human pulmonary mucus and placed into stages 2 and 3 (according to a preferential deposition of the microparticles in these stages, see Figure 5) of the next‐generation impactor (**Figure** **8**a and Figure S7, Supporting Information). Individual trajectories and mean squared displacements (MSD) of liposomes released from microparticles are represented in Figure 8b. The number of individual MSD curves with a slope > 0.5 (black line)—classifying diffusive or mobile particles—was 28.6% for plain liposomes and 33.5% for fucosylated liposomes, while a slope < 0.5 (red line) represents immobile particles (Figure 8b). The shift of the log (MSD) distribution to higher values highlights the interesting fact that mobile, fucosylated liposomes move more rapidly than plain ones (Figure 8c). However, as most liposomes are immobilized in the mucus meshwork, it is crucial to tune aerodynamic properties to avoid mucus interaction for maximizing the delivery into the respiratory bronchioles.

### 2.6 In Vitro Antimycobacterial Activity against *M. abscessus*

**Figure 9:**
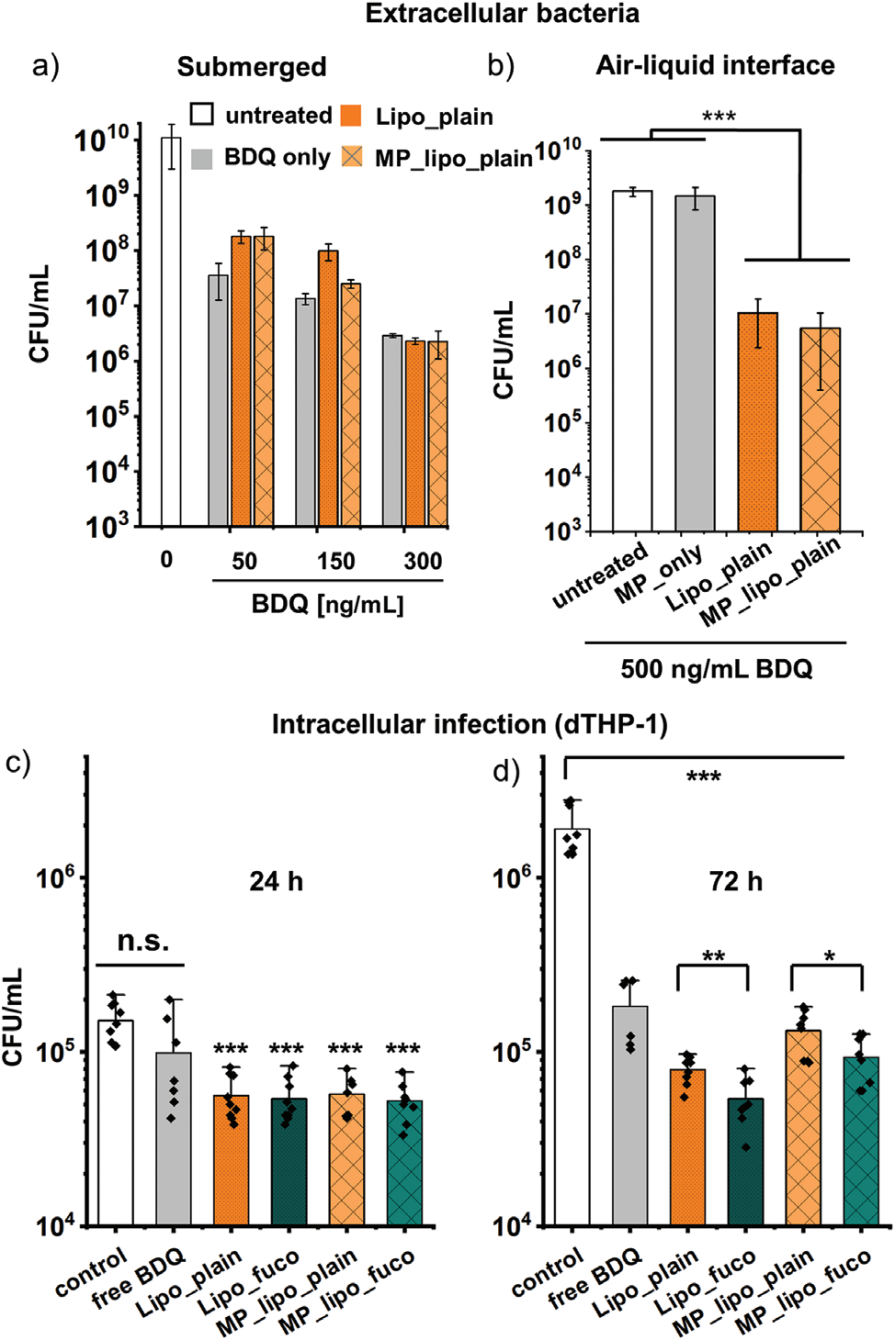
Antimycobacterial activity of free bedaquiline (BDQ), BDQ‐loaded liposomes and dry powders of BDQ‐loaded liposomes on extra‐ and intracellular *M. abscessus* under submerged and air–liquid interface conditions. a) Efficacy of the formulations against extracellular bacteria at different BDQ concentrations at submerged conditions and b) at a fixed concentration of 500 ng mL^−1^ after dry powder aerosol deposition at air‐interface conditions. Intracellular infection of dTHP‐1 cells treated with free BDQ, BDQ liposomes, and the respective powders with and without fucose targeting after c) 24 h and d) 72 h. Data represent means ± SD (*n* = 9, *N* = 3) with significance defined as *** (*p* < 0.001), ** (*p* < 0.01), and * (*p* < 0.05).

To assess the antimycobacterial activity of the dry powders and liposomes, in vitro killing assays with extracellular *M. abscessus* were performed. As the killing of extracellular bacteria does not benefit from the active targeting of macrophages, the respective experiments were performed with nontargeted formulations only. In a first step, the activity of BDQ‐loaded liposomes and microparticles was assessed at different BDQ concentrations of 50, 150, and 300 ng mL^−1^, respectively (**Figure** **9**a). At a concentration of only 300 ng mL^−1^, an almost 4‐log reduction was observed for all formulations. A similar colony forming unit (CFU) reduction was achieved when microparticles were deposited as dry‐powder aerosols from the air interface using the PADDOCC systems (cf. the Experimental Section)^[^ ^28^ ^]^ (Figure 9b), reflecting the deposition scenario relevant for lung applications. Noteworthy, due to a higher variation of the dose after aerosol deposition compared to submerged conditions—as reflected by a higher standard deviation—the amount of BDQ used in this experiment was increased to ensure sufficient deposition.

Finally, the effect of macrophage targeting on the reduction of intracellular bacteria was investigated on dTHP‐1 cells infected with *M. abscessus*. After 24 h, all formulations (except for free BDQ) significantly reduced CFU numbers (Figure 9c). Although the killing is more pronounced for liposomes before spray‐drying when compared to dry powders at the same BDQ dose, both of the targeted formulations showed improved killing compared to the nontargeted ones after 72 h (Figure 9d). In line with this finding, only when treated with BDQ, the cell viability was similar to that of noninfected cells (Figure S8, Supporting Information). After 24 h, fucosylated liposomes (Lipo_fuco) inhibited bacterial growth almost completely.

## 3 Discussion

We investigated the potential of fucosylated liposomes loaded with the second‐line antibiotic, bedaquiline, for targeting macrophages in order to improve the killing of intracellular mycobacteria. Aiming at pulmonary administration as an aerosolized medication, we further investigated the roles of tracheobronchial mucus and pulmonary surfactant in this context. A dry‐powder aerosol formulation of BDQ‐liposomes was developed by spray‐drying, yielding aerodynamic properties suitable for pulmonary delivery. While maintaining the liposomal structure and targeting ability, this formulation showed promising activity against both extra‐ and intracellular *M. abscessus*.

The delivery of liposomes to the lungs is an appealing route for noninvasive, local therapy of infectious lung diseases.^[^ ^29^ ^]^ Liposomes are versatile carrier systems that can be tuned in size, hydrophobicity, and release profile—and they can encapsulate a variety of drugs with different physicochemical properties, while surface engineering allows to specifically address alveolar macrophages, the cellular niche of mycobacterial infections.^[^ ^30^, ^31^ ^]^ Moreover, for hydrophobic (such as LVX) and poorly water soluble drugs (such as BDQ), liposomal encapsulation can solve solubility issues while reducing potential side effects including cardiotoxicity.^[^ ^32^ ^]^

Given a successful aerosol deposition in the alveolar region, the release and dissolution kinetics of liposomally encapsulated drugs are affected by the extremely small volumes and highly lipid‐rich content of the airways’ lining fluid.^[^ ^33^ ^]^ The addition of 1 mg mL^−1^ pulmonary surfactant (here applied in the form of Alveofact) to the release medium increased the amount of LVX released after 24 h by twofold until a complete release was achieved at 48 h. This is in line with a recent study with LVX‐loaded liposomes where the entire amount of drug was also released after 48 h, even though in a simple buffer (PBS, pH 7.4) only.^[^ ^34^ ^]^ In contrast, BDQ liposomes showed literally no release when Alveofact was present in the donor medium. Also in its absence, the release did not exceed 10% even after a 96‐h period. The more rapid release of LVX when compared to BDQ is likely due to the drugs’ divergent localizations within the aqueous core (LVX) as opposed to the liposomal bilayer (BDQ), respectively, thus effecting their different release profiles upon contacting a simulated pulmonary release medium.^[^ ^35^ ^]^ However, drug release upon contact with pulmonary surfactant should not necessarily be considered a constraint, since both released and encapsulated drug can act on mycobacteria that are present both intra‐ and extracellularly.^[^ ^36^ ^]^ Against this background, certain combinatorial ratios of LVX‐ and BDQ‐loaded nano‐in‐micro systems might indeed offer a “best‐of‐both‐worlds” approach as to the intra‐ and extracellular antimycobacterial eradication efficacy.

Some drug compounds, including fluoroquinolone antibiotics, can be cleared from the lungs by rapid transition across the pulmonary epithelium, which limits their pulmonary bioavailability, but increases their systemic exposure and the risk of associated adverse effects.^[^ ^18^, ^37^ ^]^ On the example of Calu‐3 cells, we demonstrated that LVX liposomal encapsulation can reduce the degree of permeation through the lung epithelium.

To improve specific liposomal uptake and intracellular routing, the addition of fucose‐derived targeting ligands is appealing because of their ability to bind to CLRs expressed on the surface of myeloid antigen‐presenting cells.^[^ ^18^, ^30^, ^38^ ^]^ Such receptors include the macrophage mannose (CD206) and DC‐SIGN (CD209) receptors present on alveolar macrophages that serve as entry ports and reservoirs of mycobacteria.^[^ ^39^, ^40^ ^]^ Following this pathway, Duran et al. demonstrated that addressing the CD206 receptor will direct fucosylated liposomes and associated antibiotics to early and late endosomes where mycobacteria typically persist. Knockout of the CD206 receptor and fucose inhibition experiments confirmed the specificity and validity of such a strategy for intracellular targeting.^[^ ^10^ ^]^ Although macrophages have a high basal phagocytic activity, the uptake into both dTHP‐1 cells and primary blood monocyte‐ and lung tissue‐derived macrophages (all of which of human origin) clearly benefitted from active targeting. Noteworthy, to observe such elevated uptake more clearly, monocytic cells were costimulated with IL‐4 to increase CD206 expression.^[^ ^41^ ^]^ Reduced uptake of fucosylated liposomes in the presence of soluble l‐fucose—i.e., a direct competitive inhibitor for receptor binding—to the level of plain liposomes confirmed the involvement of this receptor‐mediated mechanism. In addition, this pathway shuttles encapsulated drugs into compartments different from those addressed when being internalized via the phagocytic pathway. As reported recently, liposomes preferentially colocalize in endosomal compartments only after receptor‐mediated endocytosis, where they could contribute to enhanced antibiotic efficacy.^[^ ^10^, ^42^, ^43^ ^]^

None of those studies, however, have taken into account the exposure of the targeted cells to aerosolized liposomes while introducing pulmonary surfactant as an inevitable barrier to drug delivery at the alveolar interface.^[^ ^14^ ^]^ Surprisingly, when liposomes were deposited at the air‐interface (in the absence of medium), the number of cells that showed liposome uptake was higher compared to submerged conditions, while an increase in uptake of targeted liposomes was not observed. This may be related to the absence of cell culture medium, allowing a direct particle–cell contact while neglecting sedimentation effects and the particle corona formation. Sophisticated in vitro models that include relevant lung lining fluids such as Alveofact, can therefore help to elicit cell–particle interactions more closely reflecting the real‐life scenario.^[^ ^44^ ^]^ Particle–corona formation upon contact with Alveofact may influence cellular uptake, as shown in previous studies.^[^ ^45^ ^]^ In primary cells, however, a mitigated uptake in the presence of surfactant was not observed. Noteworthy, clinical surfactants including Alveofact do not contain hydrophilic SP‐A and SP‐D that are involved in host defense mechanisms and macrophage uptake while possibly underestimating the observed effects.^[^ ^46^ ^]^

When considering liposome administration via the pulmonary route, efficient deep lung deposition requires adequate aerodynamic properties. We therefore developed liposomal dry powders that coalesce high stability, propellant‐free nature, and high patient compliance.^[^ ^47^ ^]^ Spray‐drying of liposomes in the presence of the biocompatible excipients lactose and leucine yielded spherical microparticles with a homogenous surface morphology and a fine‐particle fraction of >70%, thus meeting the criteria for deep‐lung deposition.^[^ ^48^ ^]^ Whereas an aerodynamic diameter of the dry‐powder microparticles within a range of 2–5 µm is optimal for such a deposition, the size of the incorporated liposomes has to be significantly smaller. In contrast, liposomes in the size range of 100–200 nm, however, may not be optimal for efficient macrophage uptake.^[^ ^49^ ^]^ After dissolving the microparticles, the released liposomes showed a slight size increase when compared to liposomes before spray‐drying. This has been linked to structural rearrangements due to dehydration during the spray‐drying process and the subsequent rehydration.^[^ ^50^ ^]^ However, we could demonstrate that the size remained stable upon storage, and cryogenic transmission electron microscopy (cryo‐TEM) images indicated that the liposomes’ structural integrity is maintained during spray‐drying as is mandatory for retaining their targeting function.

It is assumed that a considerable portion of inhaled dry powders is trapped by steric obstruction and adhesion to pulmonary mucus covering the airway epithelium. Via mucociliary clearance, the inhaled material is expelled from the lungs, which drastically limits the retention time and local action of therapeutic aerosols.^[^ ^51^ ^]^ When placing human tracheobronchial mucus into stages 2 and 3 of the next generation impactor, we found that liposomes released from the deposited dry‐powders are sterically trapped in the viscoelastic mucus meshwork. Besides physical and hydrophobic interactions, the fact that fucosylated liposomes moved more rapidly when compared to plain ones might be due to different surface properties because of the attachment of targeting ligands via polyethylene glycol spacers. Similar observations were made by Chai et al.—i.e., one of the few studies that investigated interactions of dry powders.^[^ ^52^ ^]^ Hence, when engineered appropriately, biocompatible nano‐in‐micro formulations of BDQ‐loaded liposomes can combine both the aerodynamic advantages of microparticles and the advantages of liposomes at the cellular level while coping with the cellular and noncellular pulmonary barriers.^[^ ^53^ ^]^

A formulation optimized to reach the target site further has to prove its antibacterial activity. Thus, the antibiotic activity was first tested against extracellular *M. abscessus*—an emerging respiratory pathogen^[^ ^54^ ^]^—in the absence of macrophages. When deposited as an aerosol, microparticles showed the same activity against *M*. *abscessus* compared to a mere liposomal suspension. Subsequently, dTHP‐1 macrophages infected with M. *abscessus* were used as a model to evaluate the intracellular activity of the macrophage‐targeted formulations.^[^ ^55^ ^]^ At the early stage of mycobacterial infections, macrophages typically favor M1 polarization. However, these may develop into anti‐inflammatory M2‐like phenotypes during disease progression.^[^ ^40^ ^]^ For this reason, intracellular killing studies were performed without the addition of IL‐4.

Both liposomes alone as well as the liposomal dry powders exhibited an improved reduction in CFUs after 24 h compared to the free drug. When using TargoSphere liposomes surface‐modified with fucose‐derived targeting ligands, intracellular bacteria were killed more efficiently, most likely as a consequence of increased uptake and intracellular routing.^[^ ^10^, ^39^, ^42^ ^]^ The fucosylated liposomal antibiotic formulation essentially inhibited mycobacterial growth when comparing the CFU numbers at 24 and 72 h, which can in part be explained by the relatively slow growth of *M. abscessus* compared to other bacteria.^[^ ^56^ ^]^ Thus, although the uptake rate of nonfucosylated versus fucosylated liposomes is already evidently higher for the latter after 2‐h incubation, significant growth inhibition was only observed 72 h after treatment.

The slightly higher CFU count observed for liposomal dry powders might be related to the presence of lactose in the dry‐powder formulation; lactose was earlier demonstrated to alter mycobacterial growth and metabolism because it can serve as an alternative source for carbohydrates.^[^ ^57^ ^]^ Alternatively, a considerable fraction of extracellular bacteria present in this setup might obscure the benefit of receptor‐mediated BDQ delivery to macrophages. Finally, the effect might also result from both the presence of lactose and extracellular bacteria. The actual underlying mechanism may be clarified in future studies by using more complex models.

## 4 Conclusion

Liposomes whose surface is fucose‐decorated for CLR‐mediated macrophage targeting/uptake and loaded with the second‐line antimycobacterial drug, bedaquiline, were developed for treating pulmonary infections by intracellular mycobacteria. Spray‐drying enabled improved stability and aerodynamic properties as needed for pulmonary administration while preserving liposome size, drug load, and targeting function. Enhanced killing of intracellular *M. abscessus* was indeed observed for fucosylated as opposed to plain liposomes, suggesting that such dry‐powder formulations can combine both the aerodynamic advantages of microparticles with the fucose‐dependent targeting of nanocarriers to alveolar macrophages. Besides, the interaction with noncellular barriers such as mucus and surfactant were demonstrated as key factors that eventually could limit therapeutic outcomes. By improving the delivery to intracellular bacteria and reducing systemic drug exposure, targeted aerosolizable nano‐in‐micro delivery systems may help to cope with the ever‐increasing challenge of mycobacterial resistance.

## 5 Experimental Section

### Materials

Lactose, l‐fucose, leucine, and sodium‐fluorescein were purchased from Sigma‐Aldrich (Darmstadt, Germany). 1‐Octanol was purchased from Honeywell (Charlotte, NC). BDQ‐ and LVX were obtained from Selleckchem (Munich, Germany). BDQ‐ and LVX‐loaded liposomes (TargoSphere) were formulated by Rodos Biotarget GmbH (Hannover, Germany).

### Preparation and Characterization of Fucosylated Liposomes

Fucosylated nanocarriers used herein were CLR‐TargoSphere liposomes—i.e., one of several different lipid‐based TargoSphere nanocarriers, in this case named due to their CLR specificity^[^ ^30^ ^]^—that were loaded with either LVX or BDQ. These formulations were prepared via the thin‐film hydration method followed by extrusion as described before.^[^ ^10^ ^]^

Encapsulation efficiency and loading capacity were calculated as follows

(1) $$\text{Encapsulation}\;\text{efficiency}\;\left[\%\right]\;=\;\frac{c_{\mathrm{total}}\;-\;c_{\mathrm{out}}}{c_{\mathrm{total}}}\;\times 100$$

(2) $$\text{Loading}\;\text{capacity}\;\left[\%\right]\;=\frac{c_{\mathrm{total}}}{c_{\mathrm{lipids}+\mathrm{targeting}\;\mathrm{ligand}}}\;$$

where *c*_out_ is the concentration of the active pharmaceutical ingredient (API) in the supernatant after removing liposomes from the surrounding medium via ultrafiltration using Centrisart, and where *c*_total_ is the whole API concentration determined after the disruption of liposomes to release the encapsulated drugs into the solvent. To calculate the loading capacity, the total drug amount (*c*_total_) is referred to that of the carrier system (*c*_lipids + targeting ligand_).

Size, PDI, and *ζ*‐potential of the final formulations were determined by dynamic light scattering (DLS) using a Zetasizer ZS Series (Malvern Instruments Limited, Malvern, UK). Results are summarized in Table 1.

### Drug Quantifications

LVX‐ or BDQ‐loaded TargoSphere batches were quantified routinely for their drug loads at the Research Center Borstel according to the following method (see also the Supporting Information):

### Drug Quantifications—Quantification of LVX and BDQ via LC‐MS/MS

Liquid chromatography was performed on an Agilent 1100 Series HPLC (Agilent Technologies, Santa Clara, CA) using a SeQuant ZIC‐HILIC column (Merck Millipore SeQuant, 2.1 inner diameter × 150 mm length with 5 µm particle size, pore size 200 Å) at a column temperature of 30 °C. The mobile phase consisted of 1% formic acid (FA, solvent A) and acetonitrile (ACN, solvent B).

### Drug Quantifications—Extraction of LVX and BDQ for LC‐MS/MS

20 µL of BDQ‐ or LVX‐loaded fucosylated liposomes were diluted in 800 µL ACN and 180 µL 1% FA, followed by thorough vortexing. Afterward, 20 µL of this mixture was further diluted in 800 µL ACN and 180 µL 1% FA. The solution was vortexed again and then centrifuged for 10 min at 15.000× *g* at RT. ≈500 µL of the resulting supernatant were transferred to a 1.5 mL Eppendorf tube and recentrifuged under the same conditions. Next, 60 µL supernatant were transferred to a vial (three aliquots per sample); the injection volume was 5 µL for LC‐MS/MS analysis.

Quantifications of BDQ and LVX in dry‐powder formulations were performed as described below:

### Drug Quantifications—LVX Quantification by HPLC

LVX was quantified using a Dionex Ultimate 3000 U‐HPLC equipped with a Synchronis C18 50 × 2.1 mm, 1.7 µm column, and a UV–vis detector (all from Thermo Fischer, Dreieich, Germany). The system was operated at a flow of 0.3 mL min^−1^ with 18% of mobile phase A (acetonitrile, Sigma, Germany) and 72% of mobile phase B (0.5% trimethylamine buffer at pH 2.5). Data analysis was performed with Chromeleon 7 software (Thermo Fischer, Germany).

### Drug Quantifications—BDQ Quantification by LC/MS

Analysis was performed using an Accela UHPLC system coupled with a TSQ Quantum Access Max tandem quadrupole mass spectrometer (both from Thermo Fisher Scientific, Waltham, USA). An Accucore RP‐MS column (150 × 2.1 mm, 1.7 µm; Thermo Fisher Scientific) was eluted with mobile phase A consisting of acetonitrile + 0.1% formic acid and mobile phase B consisting of H_2_O + 0.1% formic acid. The gradient elution was applied as follows: 2 min elution at 10% A and a subsequent increase to 99% A until minute 8, which was then maintained for 3 min follow by a return to initial conditions. The system was operated at a flow rate of 0.3 mL min^−1^, with the column oven set at 40 °C. The transitions were monitored using selected reaction monitoring of BDQ (parent mass 557.15 m/z) with fragments A (58.2 m/z) and B (330.06 m/z) using heated electrospray ionization (H‐ESI) in the positive ion mode. The entire system was operated via the standard software Xcalibur (Thermo Fisher Scientific).

### Verification of the Targeting Function of Fucosylated Liposomes—LecB Binding Assay

To verify the presence of targeting ligands on the liposomes’ surface, fucosylated and nonfucosylated (control) liposomes were incubated overnight in the presence of the *Pseudomonas aeruginosa* fucose‐binding lectin, LecB.^[^ ^58^ ^]^ In brief, BDQ‐loaded liposomes were incubated overnight in PBS‐Ca^2+^ buffer (pH 7.4, 50 × 10^−6^ m Ca^2+^) at a 2:1 molar ratio of LecB/fucose‐ligand. The next day, the size of the liposomes was measured by dynamic light scattering using a Zetasizer ZS Series (Malvern Instruments Limited) and by Nanoparticle Tracking Analysis (NTA LM‐10, Malvern).

### Verification of the Targeting Function of Fucosylated Liposomes—Flow‐Chamber Experiments

To demonstrate the ability of fucose‐decorated liposomes to interact with LecB, a fluorescence intensity‐based microfluidic assay was applied.^[^ ^59^ ^]^ Plain or fucosylated liposomes (300 µg mL^−1^) in PBS/Ca^2+^ were injected into a flow chamber containing immobilized LecB for 10 min at a rate of 0.5 mL min^−1^ followed by a 30 min incubation period. Finally, to detach lectin‐bound liposomes, the chamber was equilibrated for 10 min with a l‐fucose solution (250 × 10^−3^ m). Images were acquired with a Leica DMi8 confocal microscope (Leica Microsystems, Wetzlar, Germany) equipped with a 25× water immersion objective after each equilibration step. Image processing and quantification was performed using ImageJ, version 1.52 (NIH, Bethesda, MD).

### Pulmonary Interaction Studies—Drug Release in the Presence of Pulmonary Surfactant

Prior to the experiment, LVX‐ and BDQ‐loaded liposomes were ultrafiltrated using Centrisart I 300000 MWCO (Sartorius, Göttingen, Germany) for 30 min at 2000 *g* to remove nonencapsulated drug. The release system consisted of a Slide‐A‐Lyzer MINI Dialysis Device with 10000 Dalton MWCO (Thermo Fisher, Karlsruhe, Germany) placed into a 12‐well plate (Greiner bio‐one, Frickenhausen, Germany) and filled with 3.5 mL PBS in case of LVX and PBS + Moviol 4‐88 (3% w/v) (Sigma‐Aldrich, Darmstadt, Germany) in case of BDQ. The plate was covered and placed on an orbital shaker operating at 300 rpm at RT. A volume of 150 µL of liposomes (250 and 25 µg mL^−1^ final concentration of LVX or BDQ, respectively) was added to the apical side of the dialysis device, and 200‐µL volumes were sampled at the respective time intervals from the acceptor compartment. Removed medium was replaced with fresh buffer, and the cumulative permeated mass was calculated. To study to the impact of pulmonary surfactant on the release kinetics, experiments were performed in the presence of Alveofact (Lyomark Pharma, Oberhaching, Germany) added to the donor compartment at a final concentration of 1 mg mL^−1^.

### Pulmonary Interaction Studies—Pulmonary Mucus Extraction

Mucus was collected from endotracheal tubes of patients undergoing elective surgery at the Klinikum Saarbrücken gGmbH (Saarbrücken, Saarland, Germany) according to the protocol approved by the Ethics Committee of the Saarland Medical Chamber (file number 19/15), and in line with the 2013 Declaration of Helsinki, as previously described. All patients provided written informed consent before enrollment. Only nonsmokers and patients without lung disease were included in this study. Briefly, endotracheal tubes obtained from mechanically ventilated patients were cut down to 10–15 cm pieces and centrifuged twice at 1000 rpm at 4 °C for 30 s, each, to spin down the mucus. Samples were stored at −20 °C and gradually thawed overnight prior to experimental use.^[^ ^60^ ^]^

### Pulmonary Interaction Studies—Multiple Particle Tracking in Pulmonary Mucus

A glass slide with a 10 × 10 mm gene frame chamber (Thermo Fisher Scientific) attached to the surface was placed into stages 2 or 3 of the NGI. After dry‐powder deposition, the chamber was sealed with a cover slip and imaged immediately. Measurements were performed with a Nikon Eclipse Ti‐S inverted fluorescence microscope equipped with a Nikon Intensilight 130 W mercury lamp and 40× S‐plan Fluor Nikon objective with a numerical aperture of 0.6 (all from Nikon, Tokyo, Japan). Short tracking videos of ≥20 s length were recorded at a frame rate of 20 frames per second (fps) with an Orca R2 monochrome 1.3 MP CCD camera (Hamamatsu) at a resolution of 0.135 µm pixel^−1^. 2D displacement in the X and Y directions was obtained using a ParticleTracker from *MOSAIC ToolSuite* (Plugin for ImageJ) developed by Sbalzarini and Koumoutsakos for each individual frame.^[^ ^61^ ^]^ A custom‐made *Phyton* script calculated the averaged mean squared displacement (MSD or Δ*r* ^2^(*τ*))

(3) $$\left((\Delta r^{2}\left(\tau \right)\right)=\left(\Delta x^{2}+\Delta y^{2}\right)$$

The slope (defined as *α*) of the resulting MSD allows to calculate the extent of particle diffusion within the mucus samples. We defined an arbitrary cut‐off at Ω = 0.5, thus considering particles with *α* < 0.5 as immobile and such with a slope > 0.5 as mobile within the tested mucus sample.

### Cell Culture, Isolation, and Differentiation—THP‐1 Cells

The THP‐1 human monocytic leukemia cell line (DSMZ, Braunschweig, Germany) was cultured in medium RPMI 1640 supplemented with 10% FCS during passages 8–20 at a density not exceeding 1 × 10^6^ cells mL^−1^. In a 24‐well plate (Greiner bio‐one, Frickenhausen, Germany), 200 000 cells/well at 0.5 mL were differentiated for 48 h in the presence of 25 ng mL^−1^ of phorbol‐12‐myristate‐13‐acetate (PMA, Sigma Aldrich, Darmstadt, Germany) and interleukin 4 (IL‐4, Sigma Aldrich, Darmstadt, Germany) and kept in culture for an additional 24‐h period. Differentiated THP‐1 cells are referred to as dTHP‐1 cells.

### Cell Culture, Isolation, and Differentiation—Blood MDM

Buffy coats were obtained from healthy adult blood donors (Blood Donation Center, Saarbrücken Germany) and approved by the local ethics committee (State Medical Board of Registration, Saarbrücken, Germany, permission no. 173/18). In a first step, blood mononuclear cells were separated by gradient centrifugation using Leucosep falcon tubes (Greiner, Bio‐One, Frickenhausen, Germany) in lymphocyte separation medium (Sigma, Germany). Subsequently, cells were washed twice with PBS without Ca^2+^/Mg^2+^ (Sigma Aldrich, Darmstadt, Germany) containing EDTA (2 × 10^−3^ m, Sigma, Germany). Monocytes were enriched by positive selection using anti‐CD14 microbeads (Miltenyi Biotec, USA) according to the manufacturer's protocol, and seeded at a density of 2 × 10^5^ cells/200‐µL well, and cultured in medium RPMI 1640 supplemented with 10% (v/v) FCS, 1% (v/v) glutamine and 1% (v/v) penicillin/streptomycin (P/S) for 6 d at 37 °C and 5% CO_2_. Throughout this period, cells were supplemented with 10 ng mL^−1^ human recombinant macrophage colony‐stimulating factor (M‐CSF, Miltenyi, Germany) and 20 ng mL^−1^ IL‐4 (Sigma Aldrich, Darmstadt, Germany). Medium and supplements were renewed every second day.

### Cell Culture, Isolation, and Differentiation—Alveolar Macrophages

Lung tissue was obtained from patients undergoing lung resection at the SHG Kliniken Völklingen with the consent of the Local Ethics Committee (State Medical Board of Registration, Saarland, Germany) and in line with the 2013 Declaration of Helsinki, as previously described. All patients provided written informed consent before enrollment. Only nonsmokers and patients without lung disease were included in this study. In brief, lung tissue was chopped into small pieces of ≈5 µm and washed with balanced salt solution (BSS, 137 × 10^−3^ m NaCl, 5 × 10^−3^ m KCl, 0.7 × 10^−3^ m Na_2_HPO_4_, 10 × 10^−3^ m HEPES, 5.5 × 10^−3^ m glucose, pH 7.4) with a 100‐µm pore size cell strainer (BD, Heidelberg, Germany). This filtrate mainly comprising erythrocytes and alveolar macrophages was washed again with medium RPMI 1640 (Gibco, Darmstadt, Germany) containing 5% v/v FCS and 1% v/v P/S. Next, cells were added to three Petri dishes and incubated for 1 h (37 °C, 5% CO_2_ and 95% humidity). Nonadherent erythrocytes were removed from the adherent alveolar macrophages by three washes with BSS. Cells were then cultured in medium RPMI 1640 with medium exchange after 24 h. On day 3, cells were detached with trypsin (Sigma, Germany) and counted in a Neubauer hemocytometer (Sigma Aldrich, Darmstadt, Germany) containing trypan blue for dead‐cell exclusion. A total number of 200 000 cells/200‐µL well were seeded in either 12‐well Transwell permeable supports (0.4 µm pore size, Corning Costar, Bodenheim, Germany) or 24‐well plates (Greiner Bio‐One), respectively.

### Uptake Studies—Particle Uptake

The respective macrophage variants were seeded at a density of 200 000 cells/well in 24‐well plates for submerged conditions or 12‐well Transwell plates for air–liquid interface conditions. Prior to the experiment, cells were washed with PBS without Ca^2+^/Mg^2+^, 300 µL of BDQ‐loaded liposomes (50 µg mL^−1^ in medium RPMI 1640) were added, and the cells were incubated for 2 h at 37 °C. Alternatively, medium was removed from the apical side of the Transwell, and the same liposomes were deposited with a Vitrocell cloud system (Vitrocell, Waldkirch, Germany) at a final dose of 15 µg liposomes/well in the presence or absence of 20 µL of pulmonary surfactant (Alveofact at 5 mg mL^−1^). After 2‐h incubation, cells were washed twice with PBS without Ca^2+^/Mg^2+^, and 100 µL accutase solution (Sigma) was added for 25–30 min for cell detachment.

### Uptake Studies—Flow Cytometry

Cells were transferred to FACS tubes (Greiner, Frickenhausen, Germany) centrifuged at 300× *g* for 5 min, and resuspended in PBS containing 4% FCS. Cells were measured at >10 000 cells/sample with a BD LSRFortessa flow cytometer (BD Bioscience, San Jose, CA) and analyzed with FlowJo software, version 10.7.1 (FlowJo, Ashland, OR).

### Microparticle Production and Characterization

*Production of Dry Powders*: 2.5% (w/v) of lactose solution was prepared by dissolving the lactose in Milli‐Q water by overnight stirring at 650 rpm. Leucine was added to meet a final concentration of 1% (w/v) and stirred until complete dissolution. This solution was filtered using a 0.45‐µm filter. 120 µL of BDQ‐loaded plain or fucosylated liposomes were added to the solution and gently dispersed for 5 min. The lactose microparticles used for confocal microscopy and NGI experiments were additionally stained with sodium‐fluorescein at a final concentration of 1% (w/v). The solution containing lactose, leucine, liposomes, and sodium‐fluorescein was spray‐dried using a Büchi‐90 nano spray‐dryer (Büchi, Flawel, Switzerland) under the following conditions (gas flow: 112 L min^−1^; frequency: 122 kHz, inlet temperature: 87 °C, outlet temperature: 35 °C, pump: 30%, spray: 80%, pressure: 37–38 mbar, and room humidity: 20%–30%). Produced microparticles were collected using a clean plastic scraper and stored in a desiccator at RT in the dark.

### Microparticle Production and Characterization—Scanning Electron Microscopy

Powder formulations obtained by spray‐drying were deposited on a carbon tape while applying mild airflow to remove loosely bound particles from the surface. The samples were gold‐coated (Quorum Q150R ES) and examined in a Zeiss EVO MA15 LaB_6_ field emission scanning electron microscope (Zeiss, Oberkochen, Germany) at 5.0 kV and 20 000× magnification.

### Microparticle Production and Characterization—Aerodynamic Properties of Dry‐Powder Formulations

A next‐generation impactor (NGI, model 170, Copley Scientific, Nottingham, UK) equipped with an Akita airflow unit was used to study the aerodynamic properties of the dry‐powder formulations. In brief, 10 mg of each powder sample was weighed into a clear gelatin capsule. NGI plates were coated with a 1% (w/v) polyalkylene glycol ether (Brij35) and glycerol coating solution to ensure proper particle binding to the surface of the plate upon air circulation. The capsule was placed inside a HandiHaler (Boehringer Ingelheim, Ingelheim, Germany), and the capsule was pierced once to release the dry powders. The HandiHaler was connected to a mouth piece adaptor, and pressurized air was applied for 4 s at a rate of 60 L min^−1^. Samples from all eight stages, tube, pre‐separator, and empty capsule were collected by redispersion in Milli‐Q water. The fluorescence intensity of the labeled dry powders was analyzed on a M200 plate reader (Tecan, Crailsheim, Germany) at *λ*_ex_ = 460 nm and *λ*_em_ = 515 nm. Particles in stages 2–5 were considered as a respirable portion based on their MMAD of 1–5 µm.

### Microparticle Production and Characterization—Cryogenic Transmission Electron Microscopy

Cryo‐TEM was performed on liposomes in PBS, liposomes in lactose–leucine solution, and on redispersed spray‐dried microparticles pelleted after ultracentrifugation. Briefly, 3 µL of sample was dropped onto a holey carbon grid (type S147‐4, Plano, Wetzlar, Germany) and blotted for 2 s before plunging into liquid ethane using a Cp3 cryo plunger (Gatan, Pleasanton, CA) operating at *T* = −165 °C. The sample was immersed in liquid nitrogen, transferred to a cryo‐TEM sample holder (Gatan model 914), and investigated at *T* = −173 °C by low‐dose TEM bright‐field imaging using a JEOL (Tokyo, Japan) JEM‐2100 LaB6 at an accelerating voltage of 200 kV.

### Microparticle Production and Characterization—Size and *ζ*‐Potential

Hydrodynamic diameter and *ζ*‐potential of liposomal dry powders were determined by dynamic light scattering (Zetasizer Nano‐ZS, Malvern Instruments) in PBS without Ca^2+^/Mg^2+^. 10 mg of the respective dry powders were dispersed in 1 mL of PBS and vortexed for 5 min, followed by 15 min ultrasound, and measured immediately. Measures were taken before dry‐powder production as well as after one or six weeks, respectively.

### Antimycobacterial Activity—Bacterial Culture

The *M. abscessus* smooth variant isolated from the sputum of patients with cystic fibrosis was grown for 72 h at 37 °C in 7H11 agar medium supplemented with 10% Middlebrook OADC (both from Sigma, Germany). Single colonies were inoculated into 50 mL 7H9 medium + 10% OADC and incubated for 72 h at 37 °C. Overnight cultures of *M. abscessus* were diluted (final OD 0.001) to a final volume of 100 µL in 7H9 medium and placed onto Corning Costar Snapwell permeable supports (0.4 µm pore size, Corning Costar, NY). After dry‐powder aerosol deposition (see below), inserts were placed into 12‐well plates with some plates filled with PBS to prevent evaporation. Bacteria were grown for 72 h (37 °C, 5% CO_2_, 95% RH) followed by CFU determination by dilution plating on 7H11 agar plates.

### Antimycobacterial Activity—Dry‐Powder Deposition on Cell Culture

Dry‐powder formulations were deposited using the dry‐powder deposition device on cell culture (PADDOCC) as described previously.^[^ ^28^ ^]^ In brief, 100 mg of dry powder was filled into a gelatin capsule, which then was inserted into a HandiHaler (Boehringer Ingelheim). The airstream generated by the Akita Jet System (OxyCare GmbH, Bremen, Germany) creates a pressure that releases the content of the capsule. Large particles are impacted, and the remaining smaller particles were allowed to settle for 10 min. The exposure was repeated three times to ensure a complete release of the dry powder from the capsule. The deposited amount of dry powder, as determined by measuring the fluorescence of the fluorescein labeled microparticles in the acceptor compartment, is 0.5% of the initial powder mass.

### Antimycobacterial Activity—Intracellular Infection

dTHP‐1 cells were generated as described above and infected with *M. abscessus* at a multiplicity of infection of 1:1. After 3 h, infected cells were washed carefully with HBSS (Sigma, Germany) to remove extracellular bacteria. Cells were treated with medium containing Lipo_plain, Lipo_fuco, MP_lipo_plain, MP_lipo_fuco, or nonencapsulated BDQ, at equal BDQ concentrations for 24 or 72 h, respectively. Infected but untreated cells in medium RPMI 1640 served as a control. After each time point, the cells were washed with PBS without Ca^2+^/Mg^2+^, and incubated in sterile deionized H_2_O for 30 min to lyse the cells and release the intracellular bacteria. Serial dilutions of 1:10 on 7H11 agar plates were performed for CFU determination.

### Statistical Analysis

Results were provided as means ± SD and sample sizes provided as *N* = independent experiments, and *n* = number of total measurements for all experiments with statistical analysis. One‐way ANOVA followed by Tukey's multiple comparison test was used for statistical analysis using OriginPro 2021, version 9.8.0.200. Significance was defined as ***/### (*p* < 0.001) and **/## (*p* < 0.005), unless otherwise specified.

## Conflict of Interest

The authors declare no conflict of interest.

## Author Contributions

B.C.H. designed the study, performed most of the laboratory experiments, interpreted the data, and drafted the manuscript. D.T. helped in the design of the study, performed spray‐drying experiments, and interpreted the data. A.B. and A.B. performed in vitro experiments with *Mycobacterium abscessus* and reviewed the manuscript, C.C.‐W. supervised these experiments. K.F.W.B. and C.H. produced and provided fucosylated liposomes (a.k.a. TargoSphere). R.K.G. and M.F. were involved in the data interpretation and reviewed the manuscript. F.W. and D.S. performed quantitative analysis of antibiotics. O.M. provided LecB and lectin‐immobilized flow chambers and helped in performing lectin binding assays, and A.T. supervised this work. H.H. provided lung tissue, K.S. provided pulmonary mucus, and J.H. provided buffy coats. M.K. and D.T. performed cryo‐TEM imaging. B.L. and C.M.L. interpreted data, drafted the manuscript, as well as conceptualized and supervised the work. All authors critically revised the paper for important intellectual content and finally approved the paper in the present form. All authors agreed to be accountable for their respective contribution of the work in ensuring that questions related to the accuracy or integrity of the work were appropriately investigated and resolved.

## Supporting information

Supporting Information

## Data Availability

The data that support the findings of this study are available from the corresponding author upon reasonable request.
