# Supplementary material for: Nano‐in‐Microparticles for Aerosol Delivery of Antibiotic‐Loaded, Fucose‐Derivatized, and Macrophage‐Targeted Liposomes to Combat Mycobacterial Infections: In Vitro Deposition, Pulmonary Barrier Interactions, and Targeted Delivery
Source: Adv Healthc Mater. 2022 Feb 18;11(11):2102117. doi: 10.1002/adhm.202102117 (PMC11468583; doi:10.1002/adhm.202102117)
Supplement: Supplementary file 1 — Supporting Information [file ADHM-11-2102117-s001.pdf]

ADVANCED HEALTHCARE MATERIALS

Supporting Information

for *Adv. Healthcare Mater.*, DOI 10.1002/adhm.202102117

Nano-in-Microparticles for Aerosol Delivery of Antibiotic-Loaded, Fucose-Derivatized, and Macrophage-Targeted Liposomes to Combat Mycobacterial Infections: In Vitro Deposition, Pulmonary Barrier Interactions, and Targeted Delivery

Benedikt C. Huck, Durairaj Thiyagarajan, Aghiad Bali, Annette Boese, Karen F. W. Besecke, Constantin Hozsa, Robert K. Gieseler, Marcus Furch, Cristiane Carvalho-Wodarz, Franziska Waldow, Dominik Schwudke, Olga Metelkina, Alexander Titz, Hanno Huwer, Konrad Schwarzkopf, Jessica Hoppstädter, Alexandra K. Kiemer, Marcus Koch, Brigitta Loretz and Claus-Michael Lehr**

Supporting Information to the manuscript:

Nano-in-Microparticles for Aerosol Delivery of Antibiotic-loaded, Fucose-derivatized and Macrophage-targeted Liposomes to Combat Mycobacterial Infections: *In-Vitro* deposition, Pulmonary Barrier Interactions and Targeted Delivery

Benedikt C. Huck, Durairaj Thiyagarajan, Aghiad Bali, Annette Boese, Karen F.W. Besecke, Constantin Hozsa, Robert K. Gieseler, Marcus Furch, Cristiane Carvalho-Wodarz, Franziska Waldow, Dominik Schwudke, Olga Metelkina, Alexander Titz, Hanno Huwer, Konrad Schwarzkopf, Jessica Hoppstädter, Alexandra K. Kiemer, Markus Koch, Brigitta Loretz, Claus-Michael Lehr**

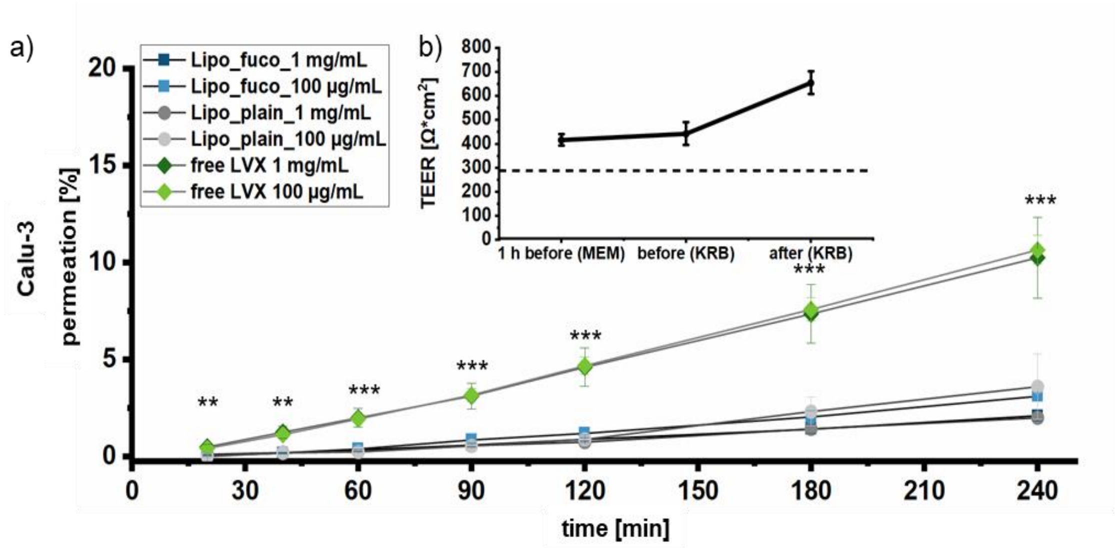

Supplementary Figure 1) a) Permeability of free and encapsulated LVX through Calu-3 cells forming a tight barrier with TEER values >400 Ω × cm^2 (b). LVX encapsulated in liposomes shows a reduced permeability and is retained by Calu-3 cells. Error bars represent mean ± SD (n = 9, N = 3)

Due to technical limitations in sufficiently solubilizing and detecting BDQ in the cell culture medium, transport experiments through Calu-3 cells (a human epithelial lung cancer cell line) were only performed with LVX-loaded liposomes *vs*. free LVX. A concentration-independent permeability of LVX through a tight epithelium (TEER > 400 Ω cm^2) was observed (**Figures S1a** and **S1b**). However, liposomal encapsulation of LVX significantly reduced its permeability. This is reflected in the apparent permeability coefficient (P_{app}), which was

0.71×10^{-6}, and 0.72×10^{-6} for targeted and plain liposomes, respectively, and 3.5×10^{-6} cm s^{-1} for free LVX at an initial concentration of 1 mg/mL. No differences were measured for targeted *vs.* plain liposomes. LVX was solely used for comparative purposes, so that the following sections will report on BDQ-loaded liposomes only.

M&M: The Calu-3 (HTB-55) human lung epithelial carcinoma cell line (ATCC, Manassas, VA, USA) was cultured in minimum essential medium (MEM; Thermo Fisher, Kandel, Germany) supplemented with 10% fetal calf serum (FCS), 1% non-essential amino acids and 1% sodium pyruvate (all from Sigma, Germany) during passages 48–53. Totals of 10,000 cells were seeded onto Transwell permeable supports (A = 1.12 cm^2) (Greiner, Frickenhausen, Germany) and grown for 12–14 days with medium replacement every second day. Experiments were performed when the transepithelial electrical resistance (TEER) values were > 400 Ω cm^2 using an EVOM2 (World Precision instruments, Sarasota, FL, USA). TEER values are determined according to:

$$\text{TEER } [\Omega \times \text{cm}^2] = R_{cells}\ (\Omega) \times A_{epith}\ (\text{cm}^2) \quad (1),$$

where R_{cells} is the cell layer-specific resistance, and A_{epith} is the area of the epithelial layer.

Prior to and after the experiments, TEER values were measured again to confirm integrity of the barrier. LVX-loaded liposomes and free LVX were suspended in Krebs-Ringer buffer at a final concentration of 1 mg/mL or 100 µg/mL, respectively, and 500 µL volumes were added to the donor compartment. At each time interval, 200 µL medium were withdrawn from the acceptor compartment and replaced with 200 µL fresh medium.

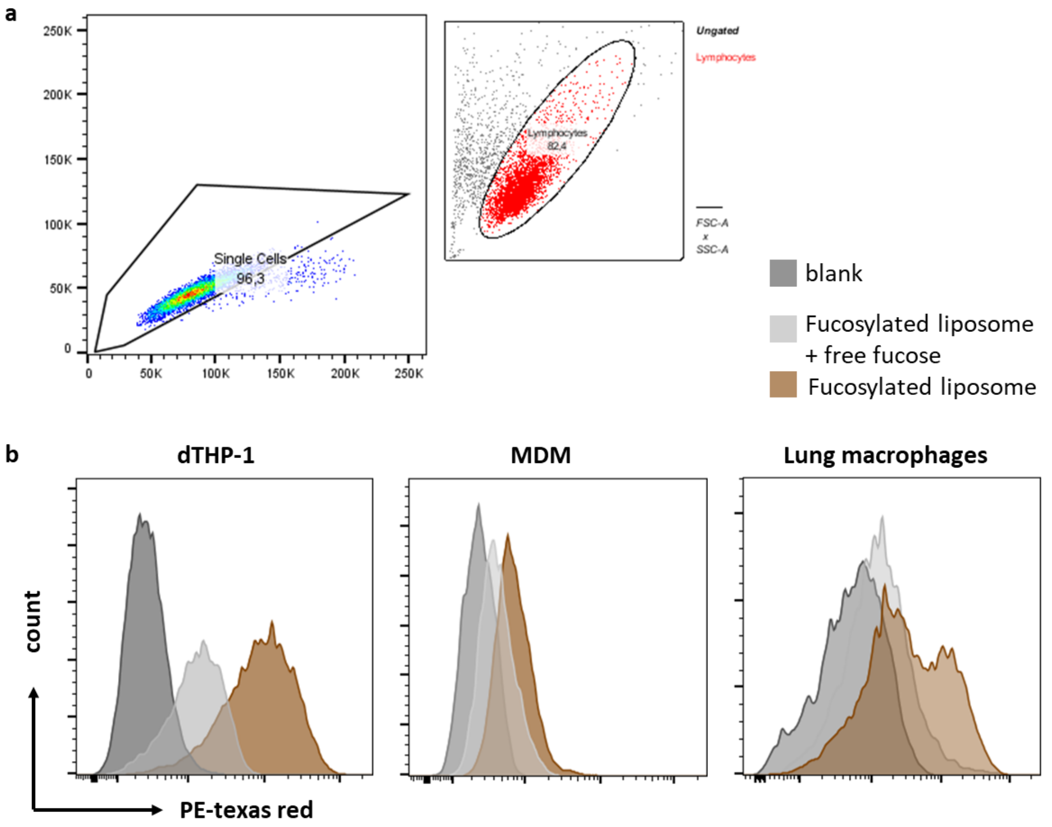

Supplementary Figure 2) a) Gating strategy for flow cytometric analysis and b) representative cyotometer plots (histograms) of phagocytic cells after 2 h at 37°C (submerged conditions) of incubation with fucosylated liposomes and in the presence of soluble L-fucose as a competitive inhibitor.

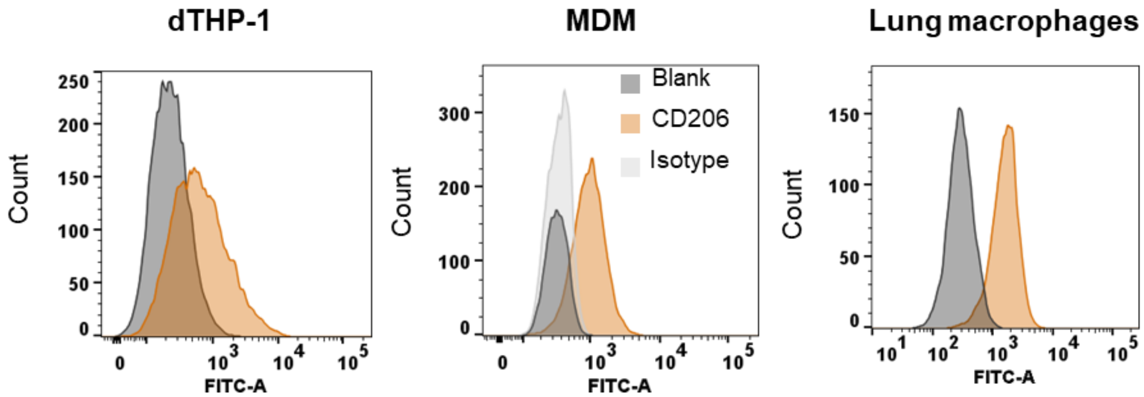

Supplementary Figure 3) Macrophage mannose receptor (CD206) expression of dTHP-1 cells, blood monocyte derived macrophages (MDM), and lung tissue-derived macrophages determined by flow cytometry.

M&M: *CD206 expression*: To determine CD206 receptor expression, cells were collected in FACS tubes at 0.5×10^6 cells /mL and blocked for 30 min with human Fc block (BD, Heidelberg, Germany). Subsequently, cells were incubated with anti-CD206 antibody (Abcam, Cambridge, UK) or isotype-matched monoclonal control antibody (Abcam, Cambridge, UK) for 2 h at 4°C in PBS containing 1% (v/v) bovine serum albumin (BSA, Sigma, Germany) and 10% FCS.

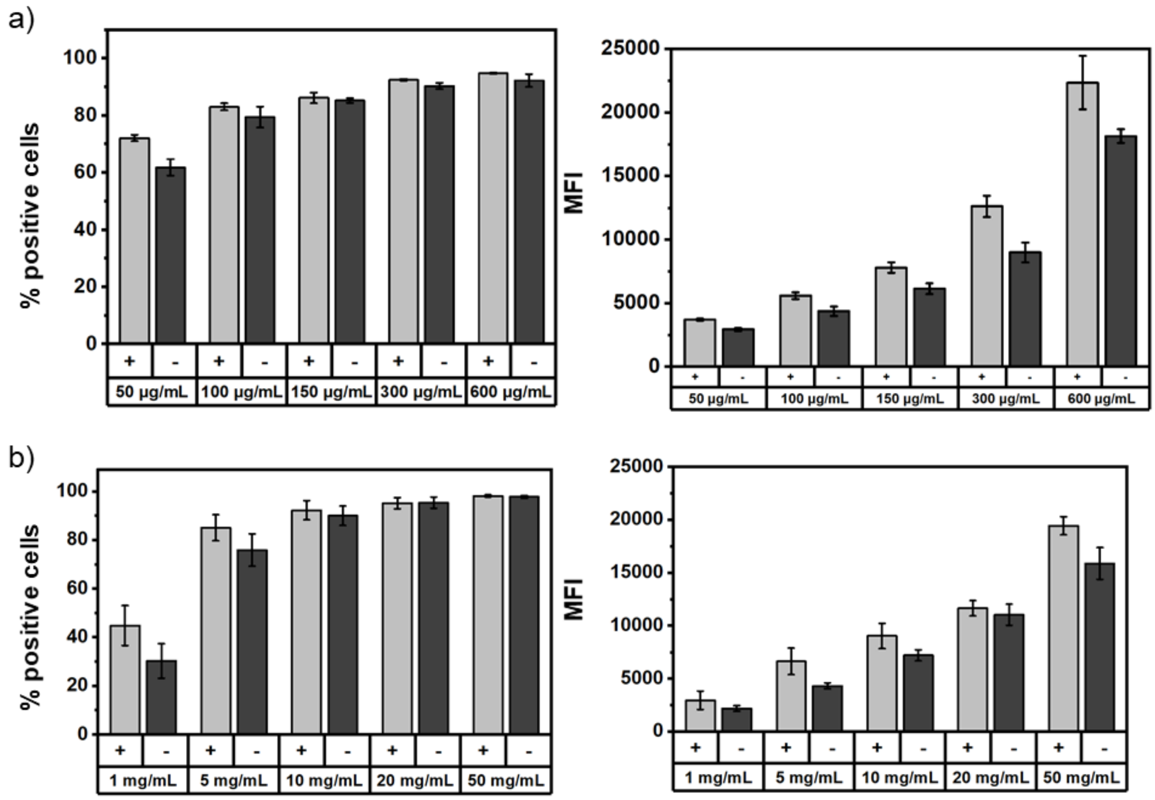

Supplementary Figure 4) a) Uptake of fucosylated liposomes (+) *vs.* plain liposomes (-) and b) the respective liposomsal dry powders at increasing concentrations denoted as µg/mL liposome or mg/mL dissolved dry powder. THP-1 cells were incubated for 2 h at 37°C and analyzed by flow cytometry.

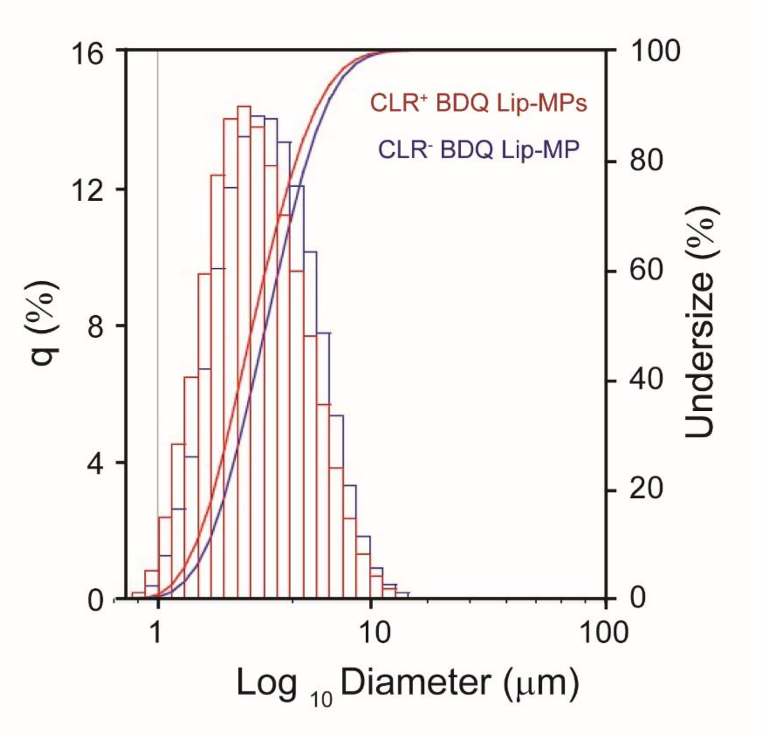

Supplementary Figure 5) Static light scattering of microparticles containing CLR-targeted (red) or plain liposomes (blue) with median sizes of 2.65 µm or 3.04 µm, respectively.

M&M: *Static Light Scattering:* For static light scattering of the particles, 15 mL of 1-octanol in a quartz cuvette served as a blank in the laser diffraction particle size distribution analyzer (Partica LA-960, Horiba). Ten mg of the powders were dispersed in 15 mL octanol, sonicated for 1 min and gradually added to the cuvette under gentle stirring. The particle size distribution was calculated as based on the reduction in transparency and scattering values. The median value of each sample was plotted.

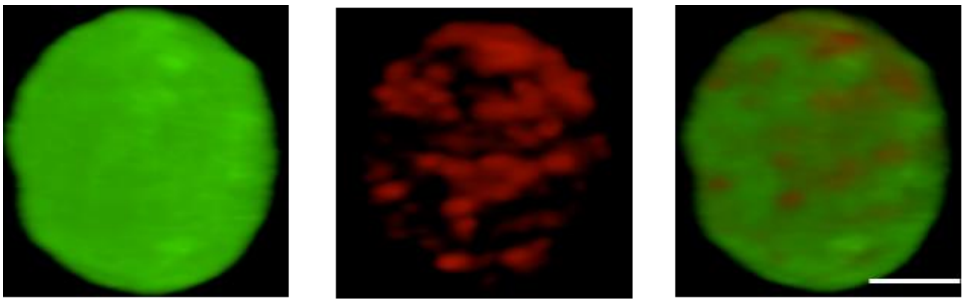

Supplementary Figure 6) Confocal microscopic micrographs of fluorescein-labeled (green) microparticles containing CLR-targeted or plain liposomes (red). Scale bar: 2 µm.

M&M: *Confocal microscopy:* One mg of the powder was admixed with 50 µL of 1-octanol and sonicated for 1 min to disperse. One microliter from the above solution was placed on a microscopic slide and sealed with a coverslip. Images were taken with a confocal microscope (Leica DMi8) equipped with a ×63 water immersion objective (HC APO CS2 63×/1.20) and image analysis was performed with LAS X software (Leica Application Suite X).

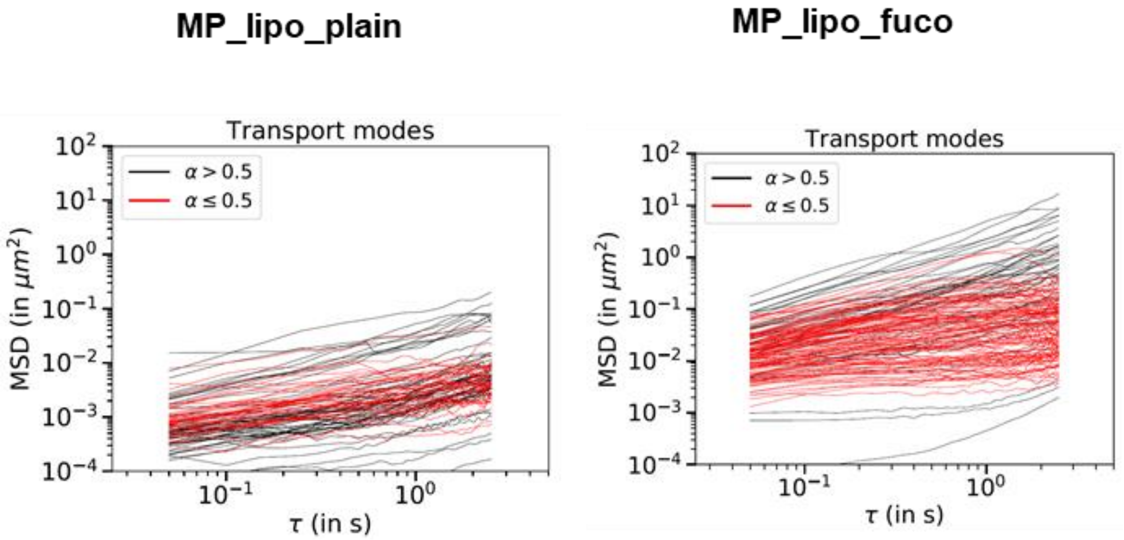

Supplementary Figure 7) Transport modes of liposomes deposited in stage 3 of the next generation impactor.

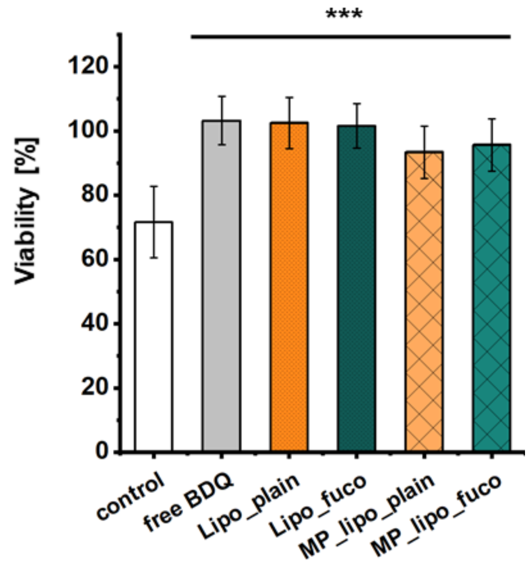

Supplementary Figure 8) Viability of dTHP-1 cells determined by LDH assay 72 h after infection. Data represent means ± SD (n=9, N=3).

M&M: *LDH assay*: The release of intracellular LDH of dTHP-1 cells infected with *M. abscessus* and subsequently treated, was measured 72 h after infection. One-hundred µL from LDH secreted to the cell culture medium (centrifuge) were transferred to a 96-well plate, and 100 µL of a mixture of 11.25 mL solution A and 250 µL solution B from LDH Cytotoxicity Detection KIT (Roche, Basel, CH) was added to the well according to the manufacturer's instructions. Untreated cells were used as a negative control; cells treated with 0.1% Triton X-100 (Sigma, Darmstadt, Germany) as a positive control; and cell-free medium served as a blank.

Cytotoxicity (LDH release) was calculated after subtracting the blank as:

$$Cytotoxicity\ (\%) = \frac{exp.value - low\ control}{high\ control - low\ control} * 100$$

Supplementary Table 1) Conditions of the hydrophilic interaction chromatography. Solvent A: 1% formic acid. Solvent B: acetonitrile.

Time [min]	Solvent B [%]	Flow [mL/min]
0.0	90.0	0.5
1.0	90.0	0.5
4.0	2.0	0.5
4.1	2.0	0.8
10.0	2.0	0.8
15.0	90.0	0.8
19.0	90.0	0.8
20.0	90.0	0.5

Supplementary Table 2) Mass spectrometry conditions including transitions of BDQ, LVX and reserpine (used as an internal standard).

Analyte	Parent [m/z]	Daughter [m/z]	Cone Voltage [EV]	Collision Energy [EV]	Dwell [s]
Bedaquiline	555.14	58.16	30	35	0.05
Levofloxacin	361.82	261.17	40	30	0.05
Internal standard					
Reserpine	608.55	194.91	30	35	0.05

M&M *LC-MS/MS***:**

Gradient: The gradient started at 90% B at a flowrate of 0.5 mL/min. After 1 min of isocratic conditions, the percentage of ACN was decreased to 2% B until minute four. At minute four, the flow-rate was increased to 0.8 mL/min. The gradient was kept isocratic at 2% B with a flowrate of 0.8 mL/min for 6 min until minute 10. Afterwards, the percentage of ACN was re-increased to 90% B until minute 15, and the flow-rate was again decreased to 0.5 mL/min until minute 19. These conditions were maintained for 1 min, so that the total run-time was 20

min (**Table S1**). The autosampler temperature was set to 4 °C, and the sample injection volume was 5 µL for LC-MS/MS analysis.

The Waters Micromass Quattro Premier XE Triple Quadrupole Mass Spectrometer (Waters Corporation, Milford, Massachusetts, USA) using electrospray ionization (ESI) was operated in the positive ion mode using multiple reaction monitoring (MRM). Parent and daughter transition was used to quantify the analytes (**Table S2**). In the mass spectrometer, BDQ, LVX, and reserpine (internal standard) were ionized through electrospray ionization source under the positive ion mode with the following source parameters: The cone gas- and desolvation gas flow were set to 100 L/h and 800 L/h, respectively. The extractor voltage was 3.0 V. We optimized the capillary voltage, the source temperature, and the desolvation gas temperature and selected 3.0 kV, 450 °C, and 90 °C. MassLynx 4.1 and TargetLynx (Waters Corporation, Milford, Massachusetts, USA) were used for operating the platform and quantifying the samples, respectively. Quantification was based on external calibration using an API-free reference as background. Stock solutions of BDQ and LVX at 0.5 mg/mL, each, were prepared in ACN. Reserpine was used as an internal standard (12.5 ng/mL in ACN) for sample processing. Calibration standards were prepared by diluting stock solutions with individual standard curves ranging from 0.00–0.005 µg/mL for BDQ and 0.001–0.25 µg/mL for LVX.

Extraction of LVX and BDQ for LC-MS/MS: Twenty µL of BDQ- or LVX-loaded fucosylated liposomes were diluted in 800 µL ACN and 180 µL 1% formic acid, and the mixture was thoroughly vortexed. Afterwards, 20 µL of this mixture was further diluted in 800 µL ACN and 180 µL 1% FA. The solution was vortexed and then centrifuged for 10 min at 15.000 × *g* at RT. Approximately 500 µL of the resulting supernatant were transferred in a 1.5 mL Eppendorf tube and re-centrifuged under the same conditions. Afterwards, 60 µL of the supernatant was transferred to a vial (three aliquots per sample), and the injection volume was 5 µL for LC-MS/MS analysis.
